# Advertisements for prescription-free drugs and dietary supplements in the Deutsche Apotheker Zeitung (German Pharmacist Journal)

**DOI:** 10.1007/s00210-024-03401-3

**Published:** 2024-09-12

**Authors:** Kristian Kuschel, Roland Seifert

**Affiliations:** Institute for Pharmacology, Hannover Medical School, Carl-Neuberg-Str. 1, 30625 Hannover, Germany

**Keywords:** Drug advertising, Medicines Advertising Law, Deutsche Apotheker Zeitung, Drug safety, Pharmacy journal, Professional journal

## Abstract

The Deutsche Apotheker Zeitung (DAZ, German Pharmacist Journal) is an independent pharmaceutical newspaper focusing on science and practice, mainly for the profession of pharmacist. In this study, drug advertising in the DAZ was analysed. To our knowledge, there is little scientific data available on drug advertising in professional journals. We assumed that professional journals provide particularly good background information on the advertised drugs because they are targeted to specialists. All non-prescription medicines and preparations that fall under the Medicines Advertising Law (Heilmittelwerbegesetz, HWG) were studied. The Medicines Advertising Law regulates the legal procedure for advertising medicinal products in Germany. The 167 product advertisements from the 52 issues of 2021 were analysed and checked for compliance with the Medicines Advertising Law. We identified significant deficiencies in compliance with the legislation. These included the lack of mandatory information required by the Medicines Advertising Law, for example the indication of adverse drug reactions and the listing of contraindications. There are very few peer-reviewed references on the efficacy of the advertised preparations. A scientific validation was carried out using the PubMed database, with the result that scientific information was available only for 1/3 of the advertisements. In addition, the appearance and target groups as well as social structures, images and feelings conveyed by the advertising were analysed. This study provides insights into the mechanisms of drug advertising in professional journals, which have not yet been researched to any great extent. Even in professional journals, pharmacological evidence plays a much smaller role than marketing, psychology and traditional social values. It seems that drug manufacturers deliberately ignore the German Medicines Advertising Law to advertise their products in the best possible way. Stricter legal controls should be put in place to prevent this practice and protect consumers from misinformation. This will increase drug safety.

**Supplementary Information:**

The online version contains supplementary material available at 10.1007/s00210-024-03401-3.

## Introduction

The Deutsche Apotheker Zeitung (DAZ) is a leading magazine for pharmacists in Germany (https://www.deutsche-apotheker-zeitung.de, last accessed November 7, 2023). Every Thursday since 1984, the DAZ informs its target group about all relevant topics regarding pharmaceutical and medical sciences, professional and health policy as well as conferences. The focus is on topics such as daily routine, counselling tips, self-medication and pharmacy practice. There are also opinions and comments from pharmacists. Many articles support the training and further education of readers. The website was launched in 1997 and includes the print version in PDF format. The journal is published by Klaus G. Brauer, Peter Ditzel and Benjamin Wessinger at Deutscher Apotheker Verlag. With around 13,000 subscribers, DAZ has the highest reader loyalty among pharmaceutical journals (Informationsgemeinschaft zur Feststellung der Verbreitung von Werbeträgern e.V., https://www.ivw.de, last accessed November 7, 2023). According to a 2017 survey, 92% of readers were very satisfied or satisfied with the magazine’s content. The advice provided and information on preparations were particularly appreciated (95%). Seventy-four percent of respondents stated that the DAZ would help them with advice recommendations directly to the customer and with the decision to purchase goods. The data comes from an in-house survey in which 1071 readers took part. (https://www.deutsche-apotheker-zeitung.de/mediadaten, last accessed November 7, 2023). The total circulation of the newspaper is 17,000 copies. By comparison, the number of pharmacies in Germany in 2022 was 18,461, of which 4743 were chain pharmacies. (https://de.statista.com/statistik/daten/studie/439697/umfrage/anzahl-der-filialapotheken-in-deutschland/, last accessed November 7, 2023).

These numbers imply that the DAZ can be found in almost every German pharmacy. It can therefore be assumed that the magazine has a major influence on the advice and recommendation behaviour of pharmacies. The content of the magazine plays a decisive role in the recommendation behaviour of pharmacists and their staff around self-medication in German pharmacies. Unlike in the USA, the Medicines Advertising Law (HWG) regulates the handling of advertising for all types of medical products, drugs or remedies. In the USA, two authorities are responsible for the advertising of medicinal products. The Food and Drug Administration (FDA) is responsible for the labelling of many products (e.g. prescription drugs, over-the-counter drugs, food) and the advertising of prescription drugs and certain medical devices. The Federal Trade Commission (FTC) regulates the advertising of food, dietary supplements and over-the-counter drugs (Aikin et al. 2016). The Medicines Advertising Law (HWG) was introduced in 1965 and aims to protect consumers (e.g. from excessive self-medication). It defines what is permitted in pharmaceutical advertising and what constitutes an offence. In Germany, the medicines advertising law consists of 18 paragraphs and an annex to §12, which deals with diseases and conditions that are excluded from advertising. In addition, the Medicines Advertising Law (HWG) distinguishes between advertising to professional circles (e.g. doctors, pharmacists or psychotherapists) and the public (consumers). (https://www.anwalt.org/hwg/, last accessed November 17, 2023).

The aim of this study was to analyse the advertising of non-prescription drugs and dietary supplements using the DAZ as paradigm and to uncover the mechanisms of advertising in professional journals. Furthermore, the compliance of the ads with the Medicines Advertising Law was examined and assessed. Attention was also paid to comparing the DAZ advertisements with those of Apotheken Umschau (Keuper and Seifert 2024). So far, there are only a few studies on drug advertising and even fewer on drug advertising in professional journals. An extensive search in PubMed revealed only few articles, but most were not relevant for this research. Even Chat GPT (show studies on pharmaceutical advertising in professional journals in Germany) found only few publications under the search term drug advertising in expert journals, all of which refer to blank pages or refer to foreign countries. Sullivan et al. (2020) reported that DTCA (direct-to-customer ads) in the USA influence the interaction between doctor and patient. They found that most respondents (76%) said they were likely to ask a healthcare provider about advertised medicines and that 26% said they had already done so. Likewise, studies from Germany report a similar picture (Kalb 2004).

## Material and methods

**Fig. 1:**
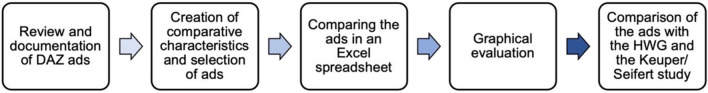
Procedure for analysing the DAZ, shown in a flow chart

All 52 issues of the Deutsche Apotheker Zeitung (DAZ) from 2021 were used and analysed. Figure 1 shows the procedure as a flow chart.

A total of 623 advertisements were considered, excluding ads for prescription drugs. After eliminating the multiple placement, 167 product ads remained to be analysed. The advertisements from the online version of the Deutsche Apotheker Zeitung (DAZ), which is identical to the print version, were documented as screenshots. In the further procedure, quantitative comparative characteristics were compiled in Excel tables. The titles, the layout, the size of the ads, the people depicted and their number, gender and age were compared. Subtle changes in multiple switching as well as associations and suggestions of the ads and the respective preparation were also detected. References to the editorial section, stated studies on the respective preparation, application category of the products, as well as stated discounts, printed vouchers and stated prices were collected. Compliance with the Medicines Advertising Law (HWG) was also checked using Excel tables. The products and criteria were entered into the table and compared. These are the mandatory details required by the Medicines Advertising Law (HWG): ingredients, indication/area of application, manufacturer, company headquarters, ADRs, contraindications, warnings and the reference to an herbal medicinal product (https://www.anwalt.org/hwg/, last accessed December 12, 2023).

Finally, the following criteria were collected and evaluated: alternative products to the preparation, pharmacy-only, incorrect content, efficacy studies, entries of the preparations in PubMed and entries of the active substance of the advertised preparation in PubMed. As a bibliographic reference database, PubMed documents medical articles in specialist journals and contains electronic references to full-text journals. The subject area includes medicine, dentistry, veterinary medicine, pharmacy, public health, psychology, biology, genetics, biochemistry, cell biology, biotechnology and biomedicine (https://de.wikipedia.org/wiki/PubMed, last accessed December 7, 2023). In the next step, the data were analysed graphically. Finally, the results were compared with the Medicines Advertising Law (HWG) and the Apotheken Umschau study by Keuper and Seifert (2024).

### Comparative features analysed

The following attributes were analysed and listed in excel sheets: title; layout; ad size; persons depicted and their number, gender and age; subtle changes in multiple placement; associations and suggestions of the ads; references to the editorial section; specified studies on the respective preparation; studies on the effectiveness of the preparation; application category of the products; specified discounts; printed vouchers; specified prices; font colour; main ad colour; size of the mandatory text; colour of the information text; legibility of the mandatory text; subjective mood in the advertisement; product category; area of application; specified manufacturer, specified trade name; registered office of the manufacturer; indication of healing promises; false content; indication of pharmacy-only status; ADRs; contraindications; warnings; alternatives to the advertised preparation; reference to a herbal medicinal product; reference to a food supplement; warning: “Risks and side effects…”; composition of the preparation and stated ingredients.

## Results

**Fig. 2:**
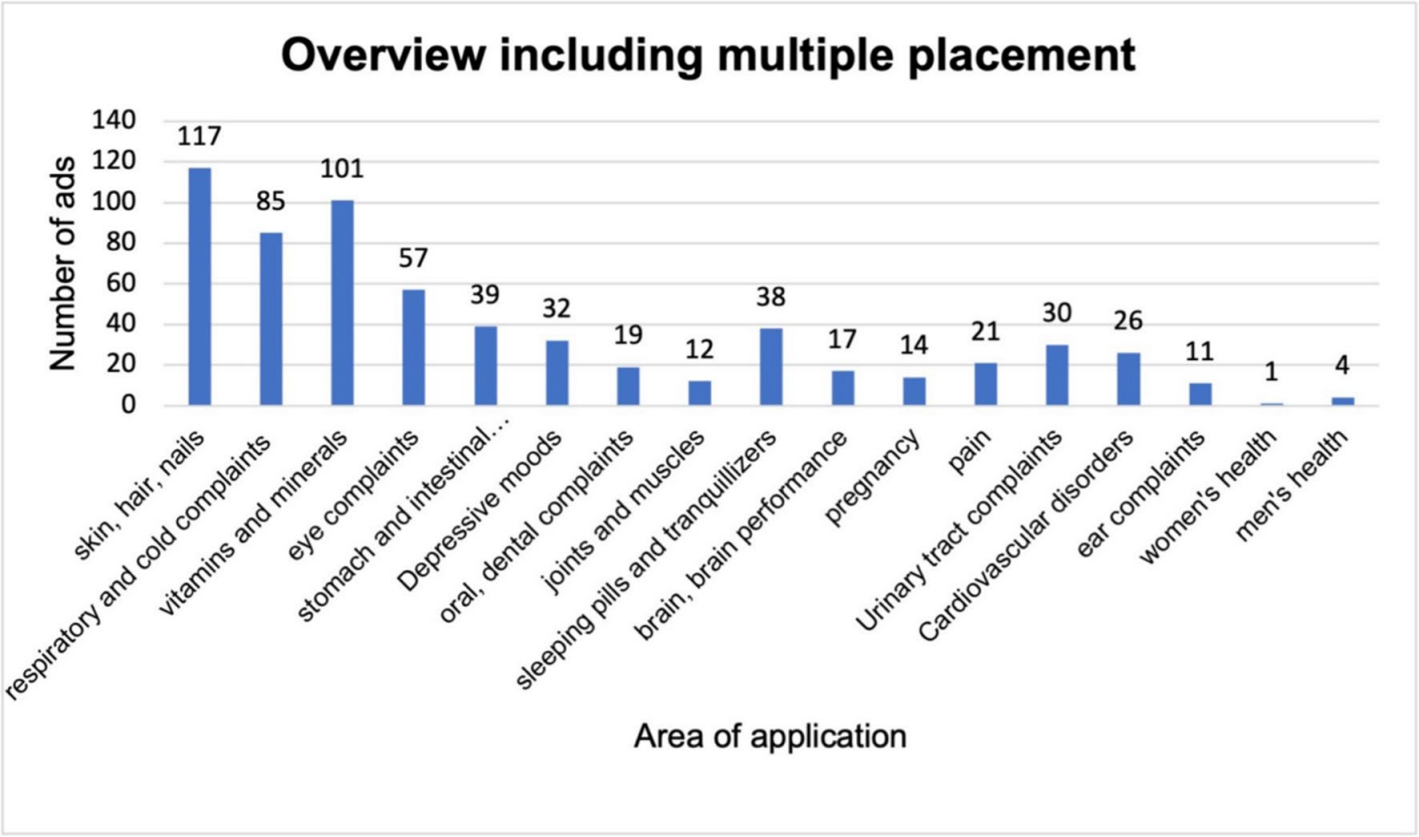
Overview of all product advertisements including multiple placement, displayed in a bar chart

**Fig. 3:**
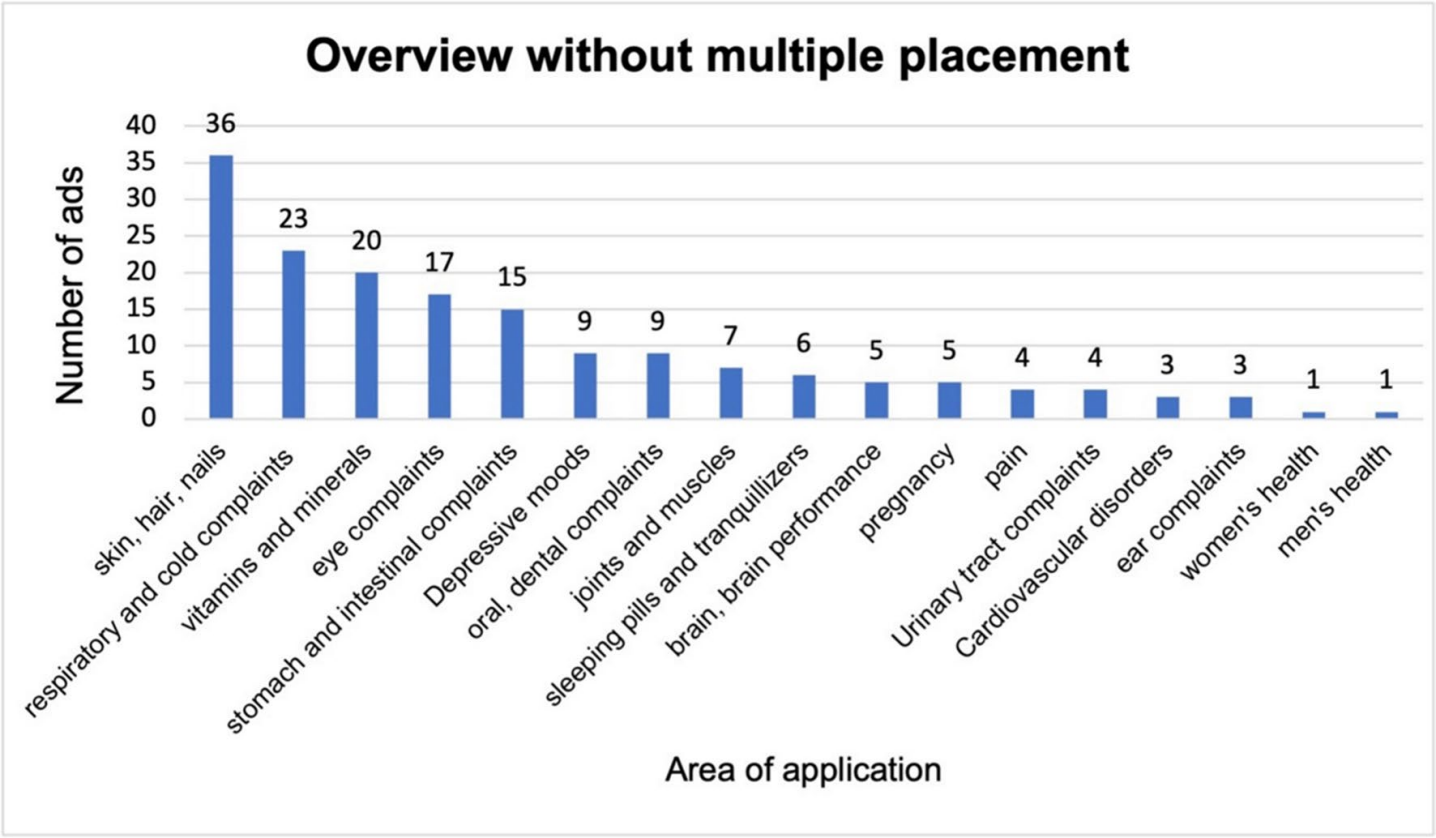
Overview of product ads sorted by frequency and area of application; the multiple placement has been adjusted here, shown in a bar chart

**Fig. 4:**
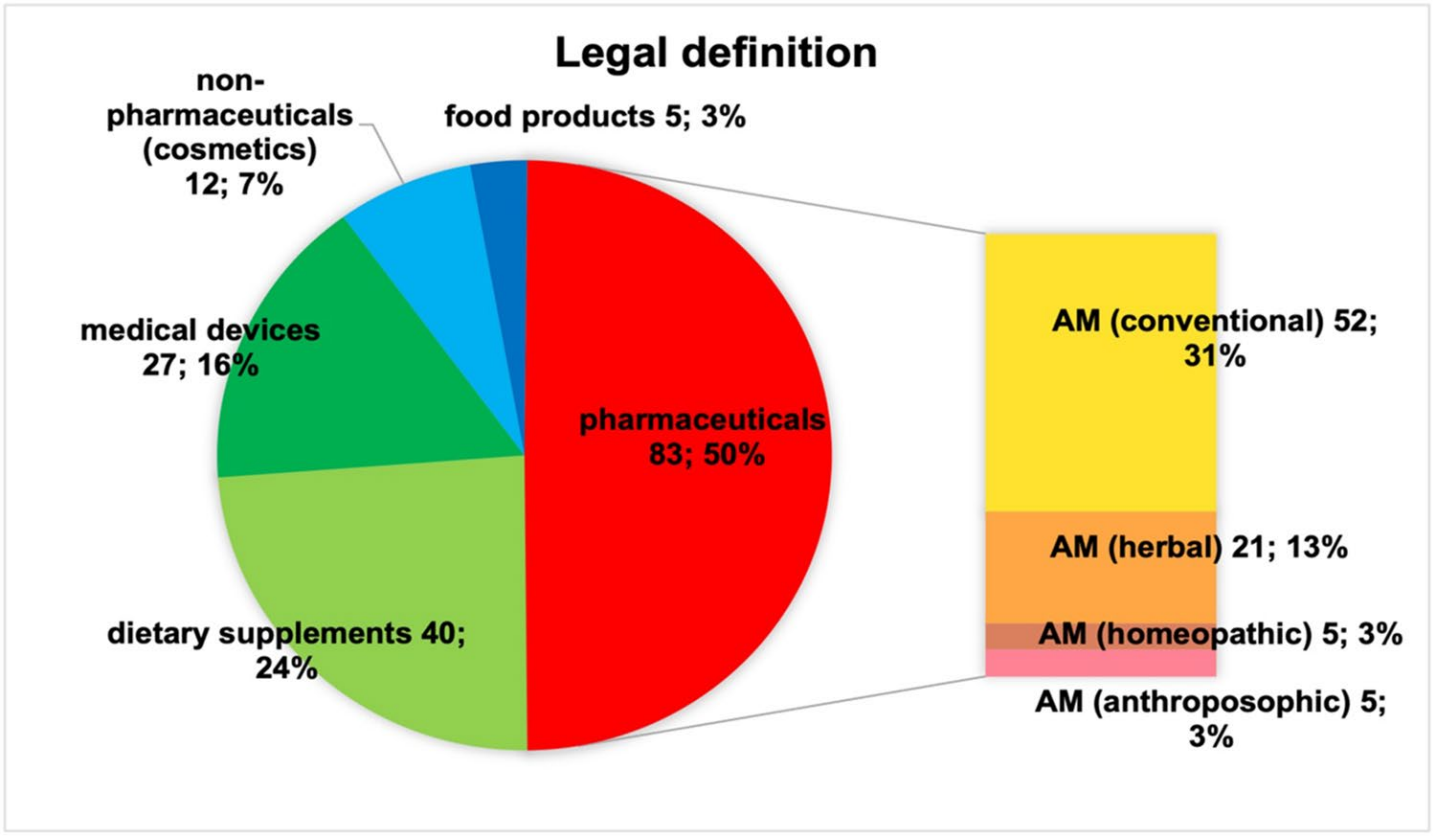
The ads categorised according to their legal definition in absolute and relative numbers, displayed as pie and bar charts. AM, Arzneimittel (pharmaceuticals)

Figure 2 shows all the product ads including multiple placement. Six hundred and twenty-three ads from 2021 were considered. There are 117 ads in the category skin, hair, nails, 101 ads in the category vitamins and minerals and 85 in the respiratory and cold complaints category. The fourth most common category was eye complaints (57 ads), followed by 39 ads for stomach and intestinal complaints, 38 ads for sleeping pills and tranquillizers, 32 ads for depressive moods, 30 ads for urinary tract complaints, 26 ads for cardiovascular disorders, 21 ads in the category pain, 19 ads for oral, dental complaints, 17 ads for the brain and brain performance, 14 ads for pregnancy, 12 ads for joints and muscles, 11 ads for ear complaints, 4 ads for men’s health and 1 ad for women’s health. Figure 3 shows the ads without multiple placement by frequency and category. The 167 ads are broken down as follows: there are 36 ads in the skin, hair and nails category, 23 in respiratory and cold complaints, 20 in vitamins and minerals, 17 in eye complaints, 15 in stomach and intestinal complaints, 9 each in depressive moods and oral and dental complaints, 7 in joints and muscles, 6 in sleeping pills and tranquillizers, 5 each in the brain, brain performance and pregnancy, 4 each in pain and urinary tract complaints, 3 each in heart, circulation and ear complaints and 1 each in women’s and men’s health. The product ads were then divided into their trade classification according to the legal definition. Figure 4 shows these categories as a pie chart and a further subdivision of the drugs in a bar chart. Fifty percent of all product ads are pharmaceuticals with 83 ads, followed by 40 dietary supplements (24%), 27 medical devices (16%), 12 non-pharmaceuticals (cosmetics) (7%) and 5 food products with a share of 3%.

**Fig. 5:**
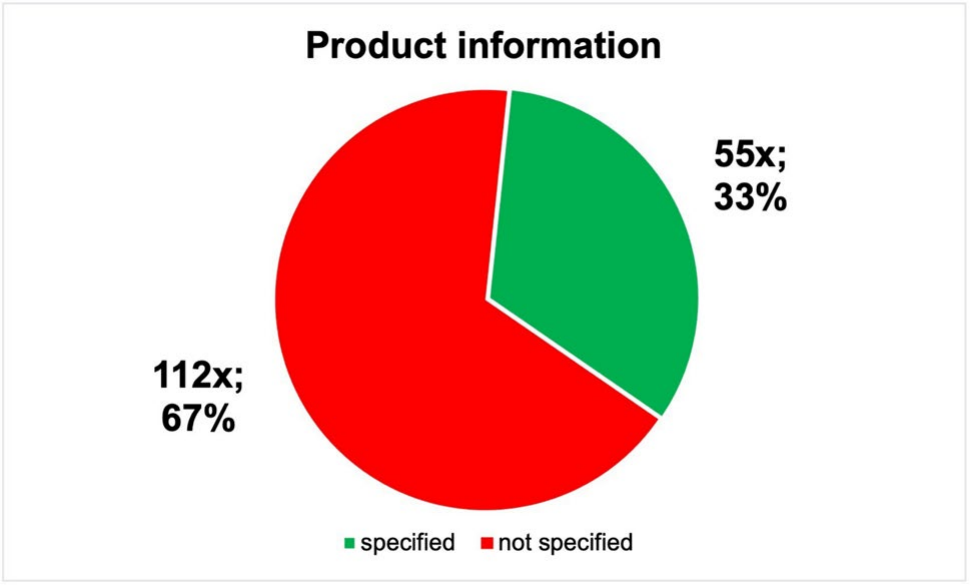
Overview of the product information in absolute and relative numbers, shown in a pie chart

The medicines were further categorised as conventional, herbal, homoeopathic and anthroposophical. This resulted in a share of 31% for the 52 conventional medicines, 13% for the 21 herbal medicines, 3% for homoeopathic medicines and 3% for anthroposophical medicines. Non-medicinal products include technical devices (e.g. wart pencils) and cosmetics. Only 55 (33%) of 167 product ads contained information on product specification (Fig. 5). In some cases, it was very difficult to find this information. Some specifications are only shown on the product package in the ad.

### Scientific validation of advertising

**Fig. 6:**
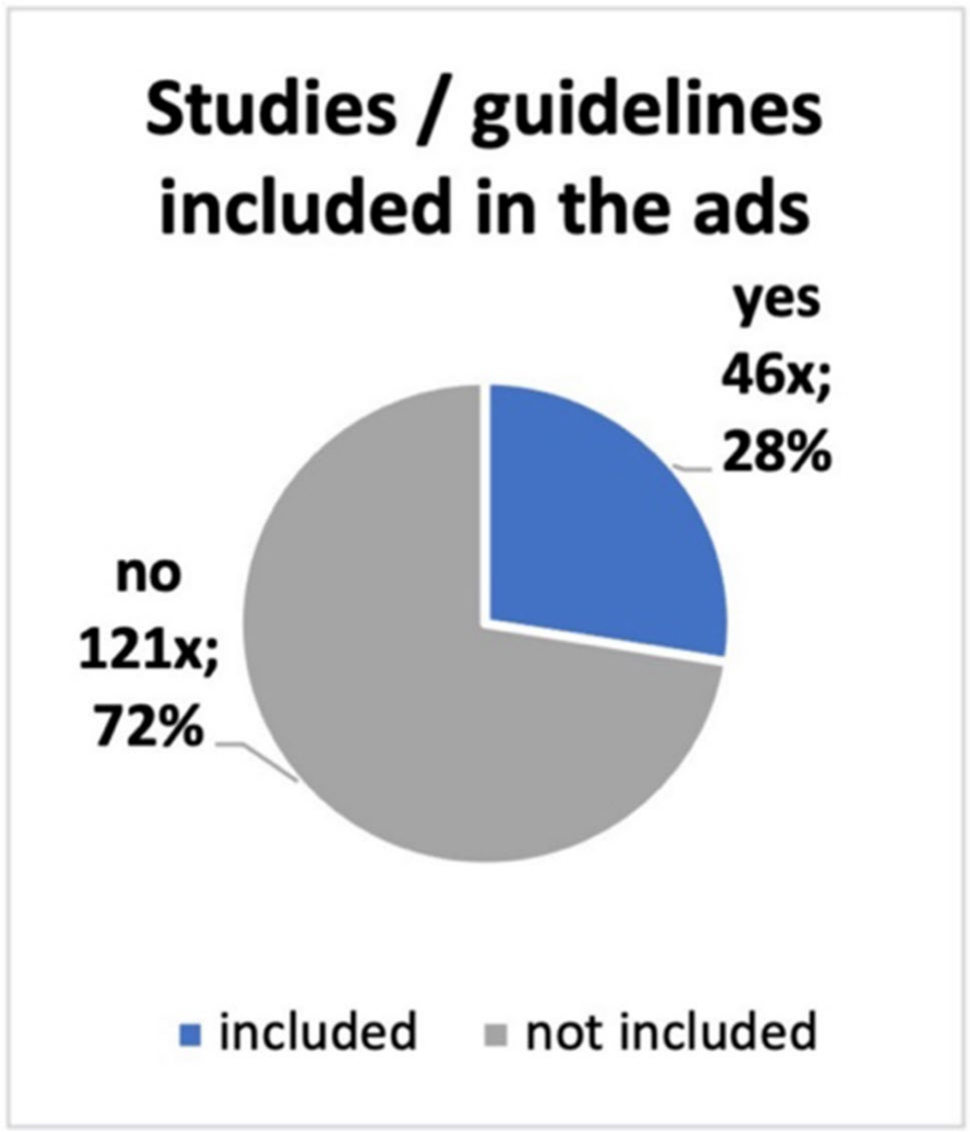
A pie chart displays the specified studies and/or guidelines included in the ads

**Fig. 7:**
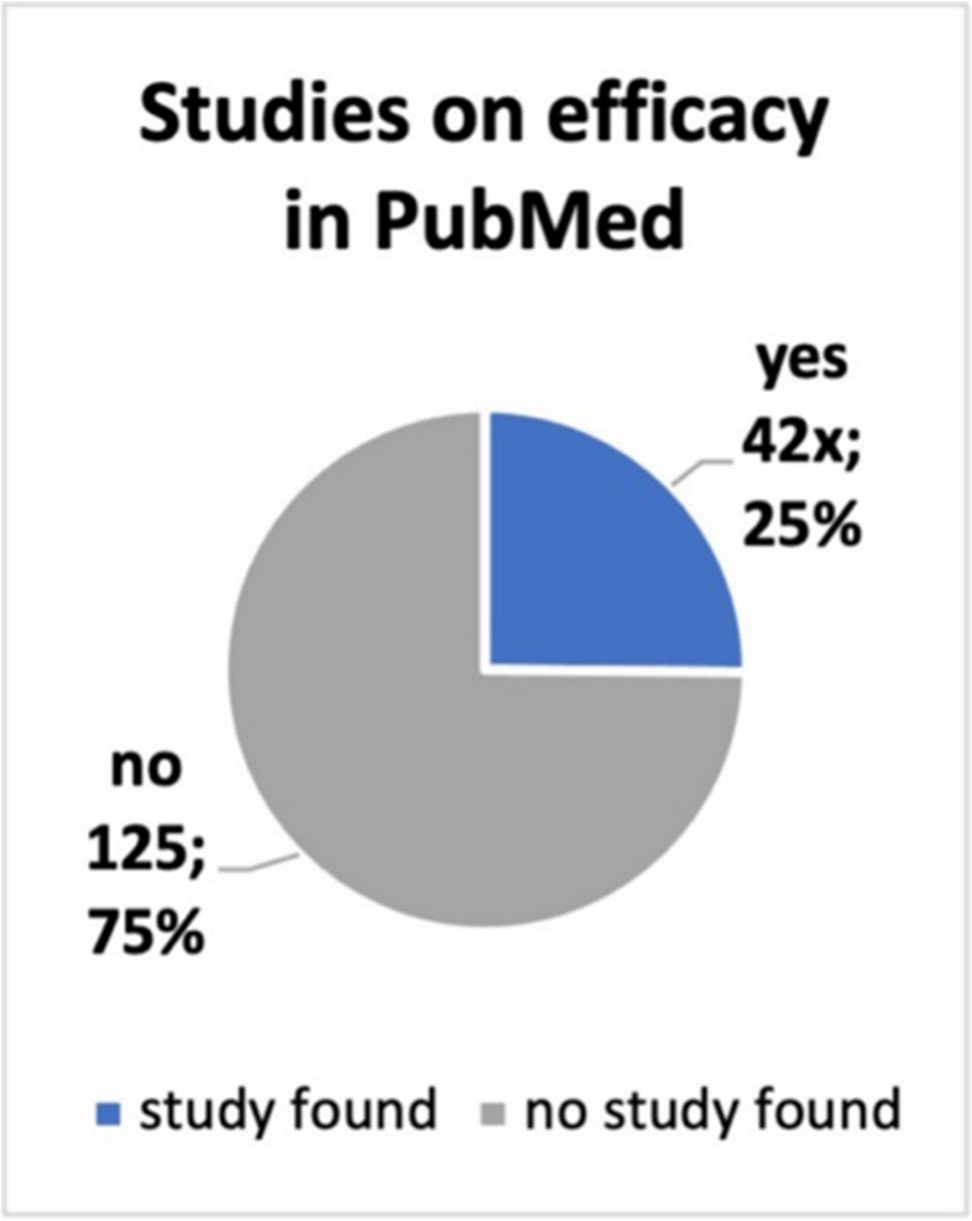
A pie shows how many studies (absolute and relative numbers) on efficacy in PubMed are included in the ads

**Fig. 8:**
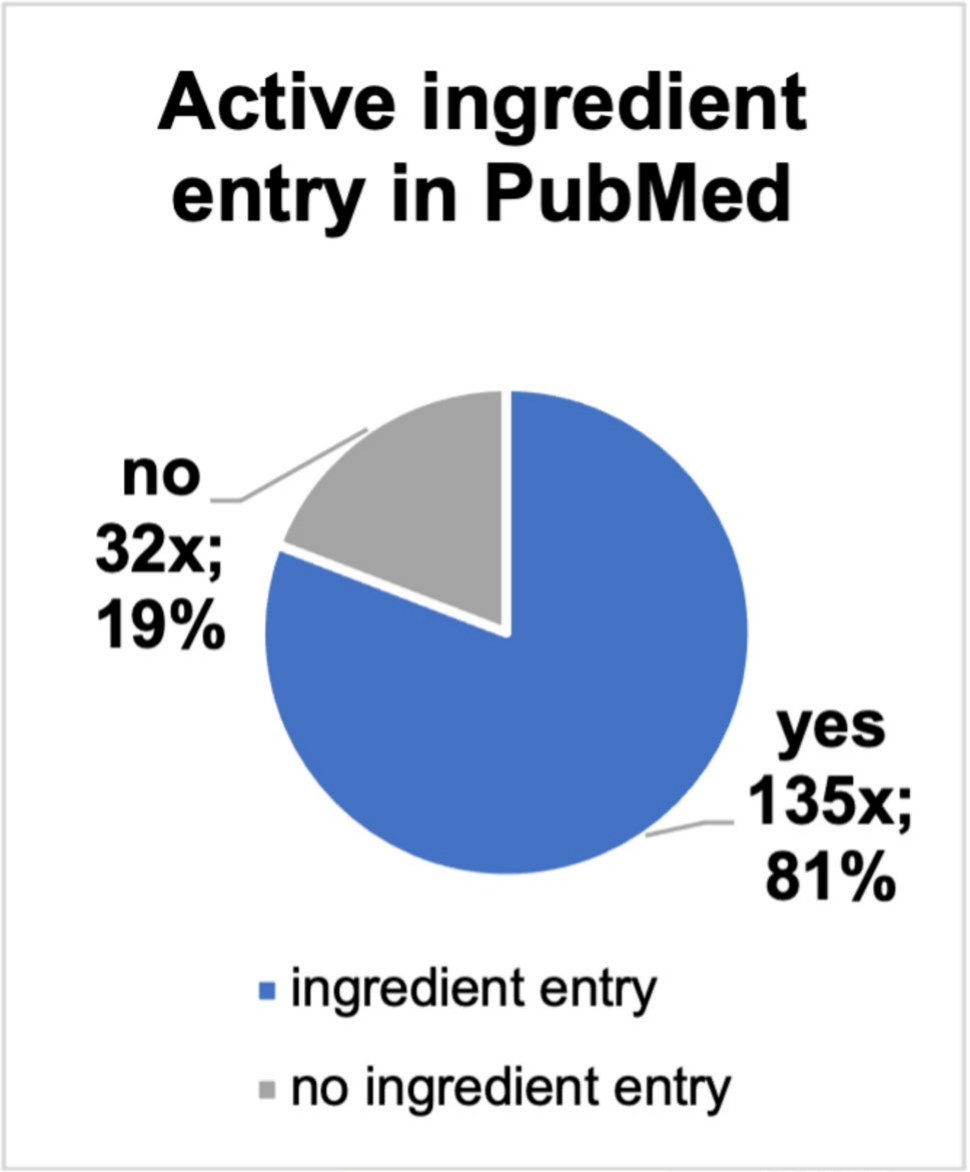
A pie chart shows how many of the main active ingredients of the preparations (absolute and relative numbers) have an entry in PubMed

The contents of the ads were checked for studies and guidelines cited in them, entries on the efficacy of the preparation in PubMed and whether there are any scientific entries in PubMed on the advertised active ingredient of the preparation*. *Figure 6 shows that only 46 of the 167 ads cited guidelines and/or studies. In 72% of the product ads, 121 times, no studies or guidelines were given*. *Figure 7 shows that a study on effectiveness can only be found for 42 advertised products (25% of the ads placed). No study could be found for the remaining 75% of the ads, which is 125 products. A search of the main active ingredients of the advertised preparations revealed that entries for 135 products could be found in the PubMed directory. No studies or entries could be found for 32 advertised preparations (Fig. 8). In lay advertising, the citation of expert opinions, certificates, scientific or professional publications and references to them is only permitted since 2012 (Huber 2012).

### Formal analysis of the advertisements

On average, there are 12 ads per magazine, of which 306 are full-page, 192 are half-page, 56 take up 1/3 and 3 take up 2/3 of a page. Twenty-two were printed on 1/4, 9 on 1/5 and 36 on 1/9. People are shown in 85 of the 167 ads (excluding multiple placement); in 82 ads, this is omitted (Fig. S1). A total of 125 people can be seen. The spread of people is shown in Fig. S2: a single person is depicted in 63 ads, two in 15, three in 4, four in 2, six in 1 and seven people in 1 advertisement. Seventy-two percent of the people (90) are women, 19% are men and 11% are children (Fig. S3). Almost no celebrities are depicted, except for Manuel Neuer (goalkeeper of the German National soccer team) in one ad. The couples depicted are exclusively heterosexual. The ethnic appearance of the people is almost exclusively European/Caucasian; only one dark-skinned person was depicted. The average age (rounded to the nearest 5 years) of all persons is 26.5 years. Most people are between the ages of 25 (40 people) and 30 (21 people). Fourteen people were children of around 5 years, 6 of around 20 years, 12 of around 40 years and 13 of around 70 years. The rest is distributed homogeneously in between (Fig. S4). The subjectively perceived mood in the ads can be divided into five categories. Fifty-four percent of the ads (90) convey a neutral mood, 39% (65 ads) a happy mood, 4% (7) a relaxed mood, 3 of the ads show a person in pain (2%) and 2 ads show an angry mood (Fig. S5). All the people in the “cheerful looking” ads were smiling. When looking at the motives underlying the ads, it was not possible to identify a focus. In 45% of the ads, the trade name of the preparations refers to the complaint or illness to be treated (Fig. S6). Ninety-two of the advertised preparations (55%) have a neutral trade name and do not allow any association or suggestion. For three preparations, the name was assigned according to the INN “International Nonproprietary Name” (https://www.who.int/teams/health-product-and-policy-standards/inn, last accessed December 17, 2023).

In one case, there is a direct reference to the editorial part of the surrounding texts (Fig. S7). The primary font colour is white in 35% of 58 ads, black in 46 ads (27%), blue in 36 ads (21%), green in 11 ads (7%), purple in 8 ads (5%), red in 5 ads (3%), brown in 2 ads (1%) and orange in 1 ad (Fig. S8 a, b). The main colour of the advertisement is green in 41 advertisements (24%), blue in 40 (24%), white in 18 (11%), purple in 16 (10%) and red in 14 (8%). Eight ads each (5%) are orange and yellow; seven ads each (4%) are primarily black, brown, and orange and one ad has the main colour gold (Fig. S9). Regarding the font size of the mandatory information and instructions, it is noticeable that they are significantly smaller than the remainder of the advertising text in the advertisements. There is no advertisment in which this text is larger or has the same size. The mandatory information is not available in 35% (58 ads). In all existing cases, the mandatory information was subjectively difficult to read (Fig. S10). The colours of the additional texts are distributed as follows: 59% black, 21% white, 11% blue, 3% grey, 3% green, 2% purple and 1% brown (Fig. S11 a, b).

### Advertisements in the focus of the Medicines Advertising Law

Table 1 shows an overview of compliance with the requirements of the Medicines Advertising Law (https://www.gesetze-im-internet.de/heilmwerbg/BJNR006049965.html, last accessed on 19 December 2023). The values marked in bold show correct compliance with the law, the values marked in italic represent aspects that are not complied with. Values marked in bold italic indicate neutral or unclear information. To obtain a good comparison with other newspapers and media, the table was created analogously to the table from the Keuper and Seifert study (2024). The first column shows the analysed criterion; the second and third columns show compliance and comments are added on the far right. The mandatory indication of the manufacturer is given in 86%; the indication of the company headquarters is given in 68%. The product name of the advertised preparation was stated in 100% of the ads. ADRs were mentioned in 44% of the ads but 56% had no mentioning of ADRs. Warnings were displayed in only 26% (Fig. S15). The ingredients of the products are stated in 68% of the ads; 32% of the ads had no indication of the ingredients. The indication and the area of application were printed in 87% of the ads, and in 13% they were missing. Excluded from this were the advertised homoeopathic medicinal products for which the indication must be omitted due to a lack of efficacy (§5 HMG: Homoeopathic medicinal products that are registered or exempt from registration under the Medicinal Products Act may not be advertised with indications. (https://www.gesetze-im-internet.de/heilmwerbg/BJNR006049965.html, last accessed on December 19, 2023). One percent of the ads referred to the statement “Ask your doctor or pharmacist about risks and side effects”, although this criterion is not a mandatory requirement for advertising in specialist circles. Nevertheless, the notice was shown in two ads. A study from the year 2000 shows that 80% of the population demand information on ADRs and contraindications (Puteanus 2000). None of the ads made a promise of a cure, which is prohibited under HMG §12.

**Table 1:** Compliance with the Medicines Advertising Law in DAZ ads. For each criterion, the percentage and, in parentheses, the absolute number are shown. For a better overview, the complied criteria are shown in bold, the non-complied criteria in italic. Bold italic indicates unclear compliance with the Medicines Advertising Law (HWG)

Medicines Advertising Law (HWG) criterion	Integrated in ad	Not integrated in ad	Comment
Specification of the manufacturer	**86% (144)**	*14% (23)*	
Specification of the company headquarters	**68% (113)**	*32% (54)*	
Indication of ADRs	**44% (74)**	*56% (93)*	
Indication of warnings, if applicable	**26% (44)**	***74% (123)***	The extent to which the warnings are necessary for the 123 ads was not checked
Specification of the composition	**68% (113)**	*32% (54)*	
Specification of the indication/area of application	**88% (145)**	*12% (22)*	The advertised homoeopathic medicines are excluded
Information on “Risks and side effects…”	***1% (2)***	***99% (165)***	This criterion does not apply here as this is an advertisement for specialised circles, but it was stated in 2 advertisements
Indication of healing promises	*0% (0)*	**100% (167)**	
Specification of the product name	**100% (167)**	*0% (0)*	
Specification of vouchers	***2% (3)***	***98% (164)***	
Indication of discounts	***4% (6)***	***96% (161)***	
Indication of prices	***1% (1)***	***99% (166)***	
Indication of “dietary supplement”	**60% (24)**	*40% (16)*	40 dietary supplements advertised
Indication of “herbal medicinal product…”	**33% (7)**	*67% (14)*	21 herbal medicinal products advertised

In 45% of the ads, the trade name suggests a mode of action or an indication. Discounts and vouchers were offered in 4% and 2% of ads. In the case of dietary supplements, 40% of the ads did not contain a reference to a food supplement. The situation is similar for the mandatory information “herbal medicinal product”, which was only referred to in 33% of cases. In 67% of the herbal medicinal products, the information was missing. In one of the ads, a price was given.

**Fig. 9:**
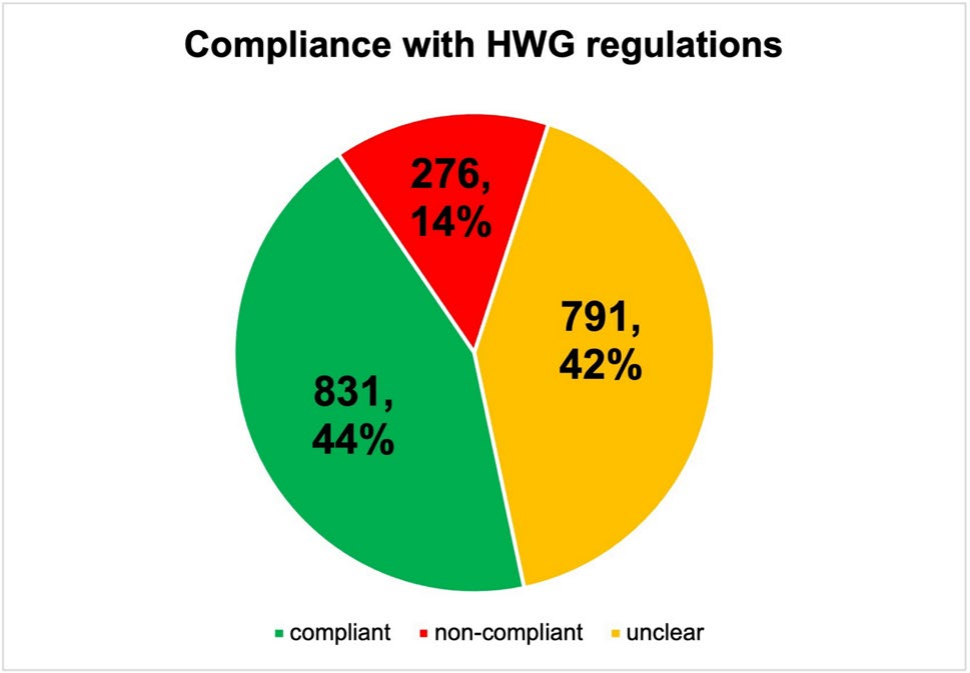
Overview of compliance with the Medicines Advertising Law (HWG) in the 167 ads analysed in the German Pharmacist Journal (DAZ), shown in a pie chart, as a derivation of the 14 criteria from Table 2. Absolute and relative numbers are shown. The difference in the total number of data points (2388 to 1898) results from the two bottom rows, as the items listed there refer to the 40 dietary supplements and 21 herbal medicinal products

For a better comparison, a pie chart was created from the data in Table 1 and compared with that of the Keuper and Seifert study (2024). Figure 9 shows compliance with the German Medicines Advertising Law in the Deutsche Apotheker Zeitung (DAZ). The data situation shows that the deficiencies in compliance with the Medicines Advertising Law (HWG) in the Apotheken Umschau are similar to those of the DAZ. The rate of non-compliance in the Apotheken Umschau is 14%, in the Deutsche Apotheker Zeitung (DAZ) 16%.

## Discussion

### Comparison of Deutsche Apotheker Zeitung (DAZ) with Apotheken Umschau

Table 2 shows the similarities/differences between the studies on pharmaceutical advertising in the DAZ and the Apotheken Umschau. In Germany, “Apotheken Umschau” is a well-known health magazine for the public that is available free of charge in pharmacies. No information on the DDD costs was provided in either journal. Most ads in the DAZ were placed in the skin, hair and nails complaint category. This category was also the most frequently advertised in the Apotheken Umschau. The second most common category (respiratory complaints) was also the same in both magazines (Fig. S12). The increased use of eye complaints could be due to the home office situation during the COVID-19 pandemic. There were 17 different products in the DAZ and 10 different preparations in the Apotheken Umschau. The DAZ is slightly ahead when it comes to citing studies on the advertised preparations, presumably because it is a magazine for specialists. In the DAZ, there was an entry in PubMed for 47 of 167 preparations (28%), and in the Apotheken Umschau, this was the case for only 23 of 123 preparations (19%). Although there are slightly more entries than in the lay press, one would expect that a professional journal provides far more scientific facts. The DAZ is also ahead in terms of the age of the people pictured, with 2/3 of the people under 40, compared with only 1/3 in the Apotheken Umschau. The ethnic classification is the same in all ads in both magazines. Almost exclusively people of European, Caucasian origin are depicted. Only one person in a DAZ advertisement had a darker skin colour. Compared to the BAMF (The Federal Office for Migration and Refugees (BAMF) is the competence centre for asylum, migration and integration in Germany) figures, 26% of the population living in Germany already had a migration background in 2019. (https://www.bamf.de/DE/Themen/Forschung/forschung-node.html, last accessed 31 Jan. 2024). There was no depiction of homosexuality in either magazine. The information on the Medicines Advertising Law (HWG) is also similar in both magazines. The company name was given in 86% of the ads in the DAZ and in 80% in the Apotheken Umschau. The composition was printed in 68% of the ads in the DAZ and 67% in the Apotheken Umschau. When analysing the information text, a similar picture emerged. In 65% of the ads in the DAZ, the information text was printed smaller than the rest of the ad. In the Apotheken Umschau, it was shown smaller in 59%. Advertised discount campaigns were found in 4% of the ads in both magazines.

**Table 2:** The differences of DAZ and Apotheken Umschau in direct comparison

	Deutsche Apotheker Zeitung (DAZ)	Apotheken Umschau (Keuper and Seifert 2024)
Pharmacoeconomic evaluation DDD costs indicated	No	No
Complaint categories and indication	1. Skin, hair, nails 2. Respiratory complaints	1. Skin, hair, nails 2. Respiratory complaints
Effects of the COVID-19 pandemic in 2021	Eye complaints (17 ads)	Eye complaints (10 ads)
PubMed entries for the advertised products	47 of 167 preparations (28%)	23 of 123 preparations (19%)
Age of the persons depicted	Approx. 2/3 under 40 years	1/3 under 40 years
Ethnic classification of the persons depicted	99% European, Caucasian origin	100% European, Caucasian origin
Depicted homosexuality	No	No
Specification of the company name	In 86% of the ads	In 80% of the ads
Specified composition	In 68% of the ads	In 67% of the ads
Size of the information text in the advertisement	65% smaller than the remaining text	59% smaller than the remaining text
Printed discount offers	In 4% of the ads	In 4% of the ads
Associations preparation name	45% suggestion by the trade name	87% suggestion by the trade name
Priority font colour in the ad	35% white; 27% black	27% white; 26% black
Emotions/perceived mood	54% no emotions; 39% happy	33% no emotions; 60% happy

### A pharmacoeconomic perspective view

The costs of the respective therapy of the advertised products remain unclear. Except for one ad, no price was given. As a result, the daily defined dose costs (DDD costs), which are the gold standard in pharmaceutical economic analyses, also remained unknown, (Ludwig et al. 2023; Trabert and Seifert 2023). Similarly, no comparisons were made with the costs of alternative therapies; this result is consistent with the data obtained for Apotheken Umschau (Keuper and Seifert 2024). It seems as if the cost–benefit structure of the preparations is not relevant and could be ignored in the advice given by the specialised pharmacy staff. It is not possible to calculate therapy costs. It remains unclear whether a cost–benefit comparison will be made at the pharmacy on request by the consumer. This fact corresponds exactly to the study by Keuper and Seifert (2024) in which the costs of the individual therapies also remained unknown. On the other hand, the products advertised here are not subject to the price maintenance obligation for medicinal products and pharmacists can calculate their own sales price (https://www.apotheker.de/apotheke/preisbindung, last accessed on January 5, 2024).

### Complaint categories and indications

The top three categories are the skin, hair, and nails, followed by respiratory, colds, vitamins and minerals. There are differences to the study conducted by Keuper and Seifert (2024) on the Apotheken Umschau. The most frequently advertised category in the Umschau was nutrition and weight loss. This category is missing in the Deutsche Apotheker Zeitung (DAZ) and no product is advertised in it. Painkillers are just as far behind in the category ranking as in the Apotheken Umschau; the argument of inexpensive medicines such as paracetamol and ibuprofen also seems to be convincing in this study. Similar to the Apotheken Umschau analysis, the categories skin, hair, nails, respiratory diseases and sleeping pills and tranquillizers are significant. The preparations in the vitamins and dietary supplements category are also frequently advertised in the DAZ and in the Apotheken Umschau. The difference to the Apotheken Umschau is the many ads placed for vitamin D preparations, which are not a focus of the DAZ. One hypothesis is that the professional associations are aware of the benefits of vitamin D supplements. As there is no proven positive effect of vitamin D in COVID-19 patients (Bignardi et al. 2023) it is therefore unlikely that pharmacies will recommend vitamin D supplements to consumers.

### Effects of the COVID-19 pandemic

The year 2021 was shaped by the COVID-19 pandemic. The extent to which the pandemic influenced the advertising behaviour of pharmaceutical companies in the DAZ cannot be mapped in this study. But with the existing study of the advertisements in the Apotheken Umschau, at least a comparison of these two journals can be made. Most of the advertisements are not in the category “sleep” as in the Apotheken Umschau, but in the category “skin, hair, nails”. The second most common category is also different in both journals. The categories heart and circulation were advertised in the Apotheken Umschau and respiratory complaints in the Deutsche Apotheker Zeitung (DAZ) (Fig. S13). The suboptimal home office conditions and associated eye complaints also analysed in the Keuper and Seifert study (2024) ((Hallman et al. 2021) and (Cartes et al. 2021)) are reflected in this study. There were even more preparations for dry eyes advertised in the DAZ than in the Apotheken Umschau. Only six preparations in the sleeping pill category were advertised in the DAZ. In contrast, 53 products were advertised in Apotheken Umschau. Furthermore, no evidence was found for the back pain thesis (Chim and Chen 2023). In the DAZ, only four different preparations were advertised in the pain category, including migraine remedies.

### Scientific principles of advertising content

Pilar Villanueva et al. (2003) analysed pharmaceutical advertising in Spain, with the result that for the 264 advertisements identified, only 125 studies were cited. Looking at the scientific basis of the advertisements placed, no studies or guidelines were given in 72% of the advertisements. Only 28% contained such a reference. After checking the products for efficacy studies in PubMed, only 25% of the advertised preparations had entries. This picture has hardly changed since 2004. A study by Tuffs (2004) analysed the advertising material of pharmaceutical manufacturers and found that around 94% of it had no scientific basis. Regarding the entries for the main active ingredients, one can find an entry for 81% of the preparations in PubMed. The extent to which the indication of the active substances matched the advertisements and whether the studies in PubMed were efficacy studies was not checked here. This shows another similarity with the Keuper and Seifert (2024) study, that scientific content is also difficult to track in the DAZ. This means that the pharmacy does not pass on any study results to help the consumer decide.

### Suggestive elements in advertising

The Deutsche Apotheker Zeitung (DAZ) mainly shows the picture of a mid-20-year-old, happy, Eurasian woman (Fig. S3 and S4). This could depend on the target group, as the readership, mainly pharmacists, should respond positively to the advertising. The women depicted are pleasant to look at, which also creates another positive effect. The other advertisements also depict people, mainly couples, mostly smiling. There are parallels here again with the Keuper and Seifert study (2024), which also shows a classically conservative image of a traditional family. As in the study just mentioned, there are no “marketing-disruptive” elements in the Deutsche Apotheker Zeitung (DAZ), such as people with a migration background or homosexuality. Despite the amendment to the Medicines Advertising Law (HWG) in the year 2012, few images of people in professional clothing (e.g. medical coats) and images of diseased body parts have been shown since then (Huber (2012).

Colours can “set positive impulses in patients” (Evans 2017), so the main colours of the advertising fonts are black (27%) and white (35%), followed by blue (21%) and green (7%) (Fig. S8). They are therefore easy to read and convey a confident look. Warning colours such as red (3%) and orange (1%) are only used to a small extent. The dominant main colours of the ads are green (24%) and blue (24%). “Green is a life-affirming colour that triggers happiness hormones”. “The physiological effect of blue is calm. The psychological effect is best described as satisfaction”. (https://www.aok.de/pk/magazin/wohlbefinden/entspannung/13-farben-ihre-psychologische-wirkung/last accessed February 28, 2024). The psychological function of green is the connection with happiness, hope and contentment. It has a relaxing effect and is intended to bring the body and mind into harmony. The psychological function of blue stands for trust, stability and harmony. It is also the most popular colour among Germans (https://www.lacke-und-farben.de/presse/pressemeldungen/die-lieblingsfarbe-der-deutschen-ist-blau) last accessed March 14, 2024). A life-affirming, calm colour is chosen in over 50% of the ads (Fig. S9 a, b).

The name of the preparation was also analysed. It was checked whether the trade name of the preparation indicated the complaints to be cured. The names of 45% of the preparations of the advertisements referred to the disease to be treated. Compared to the Keuper and Seifert study (2024), this is 42% less (Fig. S6). The gold standard in professional pharmacology is the INN, the international non-proprietary name according to the WHO, which was chosen in only three preparations. In almost all advertisements, the preparations are shown as OVP, presumably so that pharmacy staff memorizes the packagings and are thus able to pick them up from the shelf more quickly during the consultation. There is a reference to the editorial section in only 1% of the advertisements (Fig. S7). It is common in print advertising for an editorial section to refer to the advertised product. This procedure is not used in the DAZ. One attempt to explain this could be that the advertised preparations have been repeatedly advertised over the years and no longer provide any new pharmacological topics. For comparison, editorial texts were written in Apotheken Umschau for 3% of the advertised preparations. The difference here is the readership, as the DAZ is for specialists, and “trivial” articles could not find a place here.

### The Medicines Advertising Law (HWG)—insufficient compliance

Compliance with the Medicines Advertising Law (HWG) is one of the focal points of this study. The extent to which the legal requirements are complied with in the DAZ advertisements was reviewed. In addition, the comparison of compliance with the Medicines Advertising Law (HWG) with the Keuper and Seifert study (2024) is included here.

Adverse drug reactions and contraindications were stated in just under half of the advertisements. ADRs were mentioned in 44% of the advertisements (Fig. S13); contraindications were mentioned in 46% of the advertisements (Fig. S14). It is questionable whether pharmacists/pharmacy staff are expected to know the ADRs or whether they are to be displayed by modern computerised cash register systems and have thus been eliminated. The extent to which this information is mandatory for healthcare professionals could not be conclusively examined here, partly because the healthcare professionals were expanded at the end of 2023, and nursing staff are now also included in the healthcare professionals (Knierim 2023). Another point is the possible deterrence of potential customers by reading the ADRs and contraindications. There is also a parallel to the Keuper and Seifert (2024) study in this point; in Apotheken Umschau, these criteria were considered in only 2% of the advertisements. It can therefore be confirmed that the omission of the mandatory information in Apotheken Umschau most likely serves to promote sales.

The composition was stated in 68% (Fig. S12), similar to the Keuper and Seifert study (2024), in which 76% of the ingredients were listed. The situation is similar regarding the mandatory warnings, which were printed in 26% of the advertisements (Fig., p16), compared to the Apotheken Umschau which stated 27%. The indication of the company name in the advertisement is mandatory according to the Medicines Advertising Law and was indicated in 86% (Fig. S16); by comparison, in the analysis by Keuper and Seifert (2024) in Apotheken Umschau, the company name was indicated in 80% of the advertisements. The hypothesis questioned there that omitting the company name would avoid communication with the public could not be confirmed, as the difference between the two values is 6%. Furthermore, the company headquarters was printed in only 68% of the advertisements (Fig. S19). The product name was mentioned in all advertisements.

In contrast to the study by Keuper and Seifert (2024), the reference to “Risks and side effects…” is not relevant in this analysis, as it is not required for advertising for healthcare professionals. Nevertheless, this text was printed in two advertisements in the DAZ (Fig. S17); presumably, these advertisements are identical to those placed in the lay press. They were probably not adapted for the professional magazine. Only 33% of the individual product category information in the advertisements was implemented (Fig. 5). The resulting mixture of food, dietary supplements and medicines should also be viewed critically. Thus, dietary supplements are advertised in one go with medicines in visually almost identical advertisements. The legal requirements for the various products are not or only insufficiently considered (Trabert and Seifert 2023). False statements were made in 4% of the advertisements placed (Fig. S18). For example, the advertisements referred to internal cleansing, one statement read: “No germs get in” and one advertised a restoration of the immune system. These statements are to be viewed extremely critically, as they are clearly prohibited by the Medicines Advertising Law §3 (https://www.gesetze-im-internet.de/heilmwerbg/BJNR006049965.html, last accessed on February 28, 2024).

This analysis clearly shows that there are no effective control mechanisms for ad placement. The pharmaceutical companies can apparently place any ad they want. The Deutsche Apotheker Verlag, the publisher of DAZ, is being paid for the ads. This creates a classic conflict of interest that shifts pharmaceutical advertising into a grey area. Thus, stricter control and enforcement of the HWG would probably result in fewer advertisements being placed.

### Limitations of the study

Despite the care taken in conducting the analyses, there are some limiting factors. All advertisements for non-prescription products from 2021 were analysed. The analyses and results of this study are based exclusively on information from the publicly available advertisements and public information on the Medicines Advertising Law (HWG) and the respective manufacturers. In addition, the review of compliance with the Medicines Advertising Law (HWG) was not carried out by lawyers, but by authors trained in pharmacy, medicine and pharmacology to the best of their knowledge and belief. In addition, this study deals with advertising for professional circles, which means that it is not always possible to determine whether some features violate the Medicines Advertising Law (HWG). The formal analysis of the advertisements is also somewhat subjective in terms of mood and legibility.

### Suggestions for future studies and conclusions

The study, like that on Apotheken Umschau by Keuper and Seifert (2024), shows that the Medicines Advertising Law is only insufficiently complied with. Marketing and strategic factors outweigh the pharmacological content of the advertising even in professional journals. Furthermore, it is obvious that compliance with the Medicines Advertising Law (HWG) is not being controlled. Stricter monitoring by the federal authorities would be advisable to increase drug safety.

Future studies could investigate drug advertising in other media. TV magazines, daily newspapers, TV stations, social media and radio ads would be suitable. By analysing the major media, the deficits of pharmaceutical advertising would become even more evident and render it possible to make important changes for the consumer to increase drug safety. This is because the trivialisation of preparations that are not subject to prescription is a serious risk (Taylor and Davies 2018, McCrae et al. 2018). With the increasing use of vitamin D treatments, the number of vitamin D poisoning cases has also increased significantly. Many of these cases are due to inappropriate prescribing and the use of high-dose over-the-counter or unauthorised preparations.

Another danger is the masking of symptoms of over-the-counter medications and the risk of potential interactions between prescribed and over-the-counter drugs (Parvez and Rishi 2019, Scherf-Clavel 2022). The same applies to concealing the use of over-the-counter medicines and food supplements from the treating doctor, sometimes by not clearly labelling the preparations. A start would be a clearly defined scheme for pharmaceutical advertising, including a clear indication of which products are involved, e.g. medicines and dietary supplements. Such a scheme could be used to compare the mandatory information by using automatic text recognition. The content of the advertisement would probably still have to be checked manually, as automated systems cannot evaluate the special pharmacological statements.

Another approach would be to impose a shared responsibility on magazine/journal publishers. This could minimise the conflict of interest that arises from paying for the placement of advertising. The publisher would then have to point out at least the simple formal compliance requirements such as company name and registered office. It is important that doctors and other healthcare professionals critically analyse and review the information they receive from such advertisements to ensure that they are making informed decisions in the best interests of their patients. An alternative control body would be the review of regulations by pharmaceutical competitors. This would shift control to the warning letter system in Germany. Unfortunately, this has recently only been practised with the gendering of the mandatory text “on risks and side effects…”, (Tröbitscher 2023). Other professional journals such as the Deutsches Ärzteblatt (German Physicians Journal) should be analysed in further studies to obtain a more comprehensive overview. It will also be important to analyse drug advertisements in professional journal appearing in other countries.

### Take home messages

This case study on the Deutsche Apotheker Zeitung (DAZ) has revealed several new aspects of pharmaceutical advertising in professional journals:

- Scientific evidence plays a subordinate role in drug advertising in professional journals.
- Marketing and psychology dominate pharmacological evidence.
- Pharmacoeconomic information is not provided.
- The social image conveyed is traditional and very homogeneous to ensure a positive marketing climate.
- Several important aspects of the Medicines Advertising Law are only incompletely fulfilled or not fulfilled.
- There is indirect evidence for a conflict of interest of magazine publishers with pharmaceutical companies placing ads.
- The principles governing drug ad placement in magazines for lay persons and professionals (here pharmacists) are surprisingly similar.

## Supplementary Information

Below is the link to the electronic supplementary material.

Supplementary file1 (DOCX 1.95 MB)

## Data Availability

All source data for this study are available upon reasonable request.

## Abbreviations

DAZ: Deutsche Apotheker Zeitung (German Pharmacist Journal)
HWG: Heilmittelwerbegesetz (Medicines Advertising Law)
ADRs: Adverse drug reactions
AM: Arzneimittel (pharmaceuticals)
INN: International Nonproprietary Name
OVP: Original packing
DDD: Daily defined dose
