# Supplementary material for: Advertisements for prescription-free drugs and dietary supplements in the Deutsche Apotheker Zeitung (German Pharmacist Journal)
Source: Naunyn Schmiedebergs Arch Pharmacol. 2024 Sep 12;398(3):2739–51. doi: 10.1007/s00210-024-03401-3 (PMC11920363; doi:10.1007/s00210-024-03401-3)
Supplement: Supplementary file 1 — Supplementary file1 (DOCX 1.95 MB) [file 210_2024_3401_MOESM1_ESM.docx]

**Supplemental Figures S1 – S19**

**Kristian Kuschel and Roland Seifert**

**Advertisements for prescription-free drugs and dietary supplements in professional journals: A case study on the Deutsche Apotheker Zeitung (German Pharmacy Journal)**


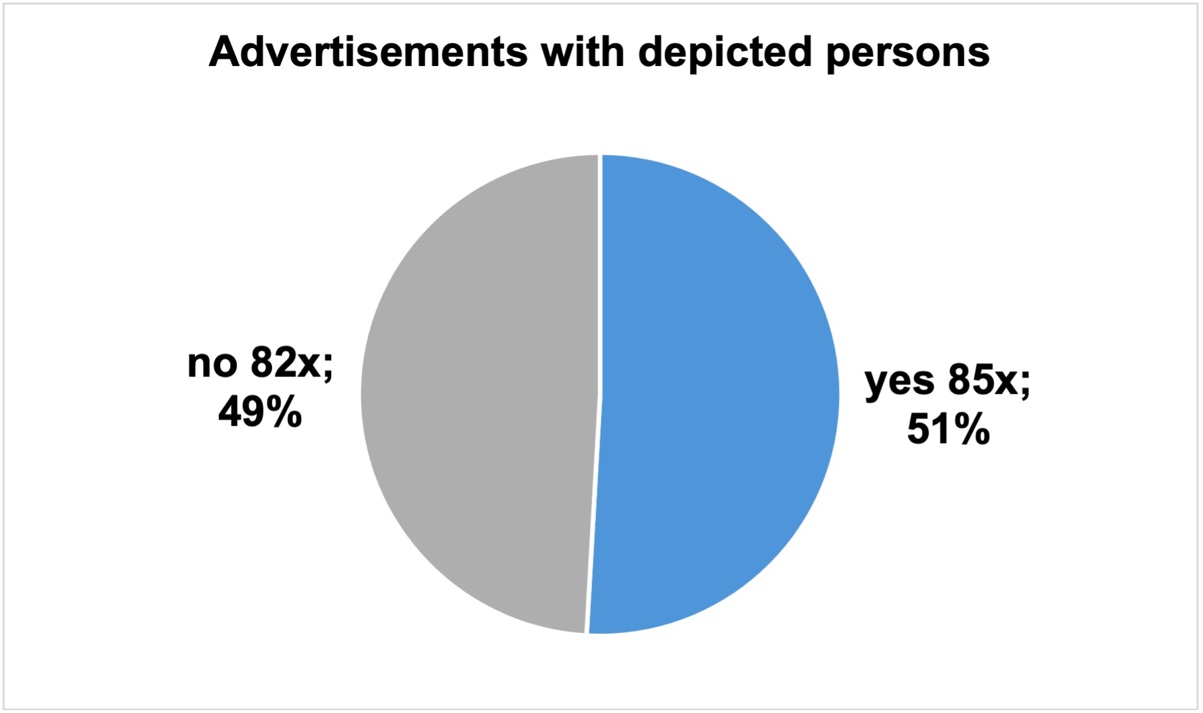


**Fig. S1** shows the people depicted in the advertisements in the pie chart as a percentage and in absolute terms


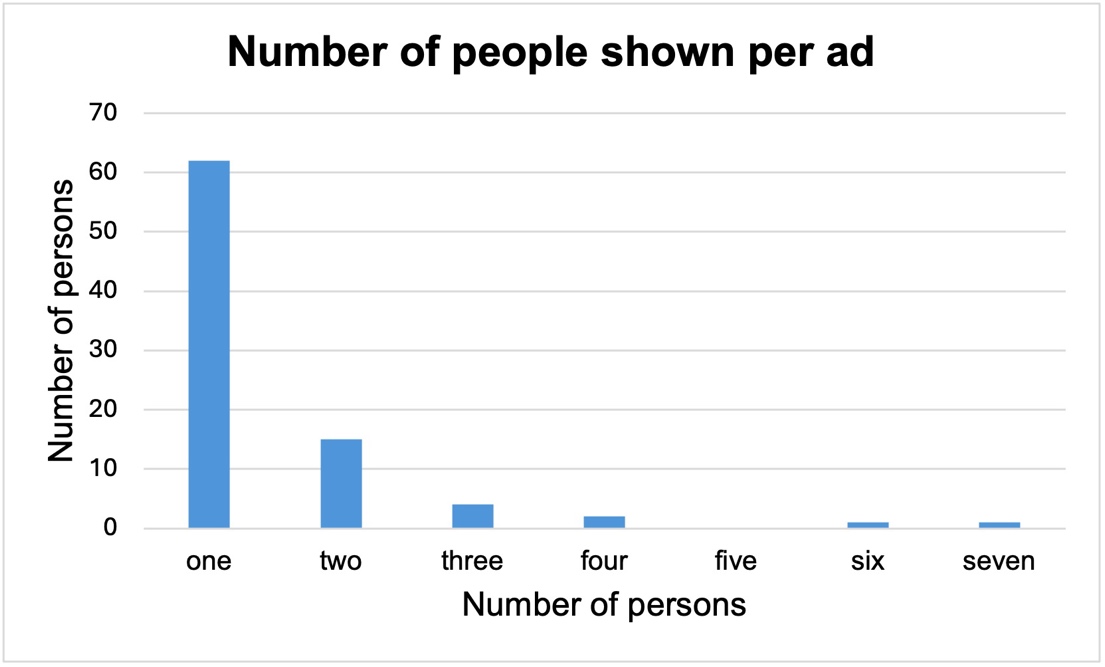


**Fig. S2** shows the people shown per ad in a bar chart as absolute numbers


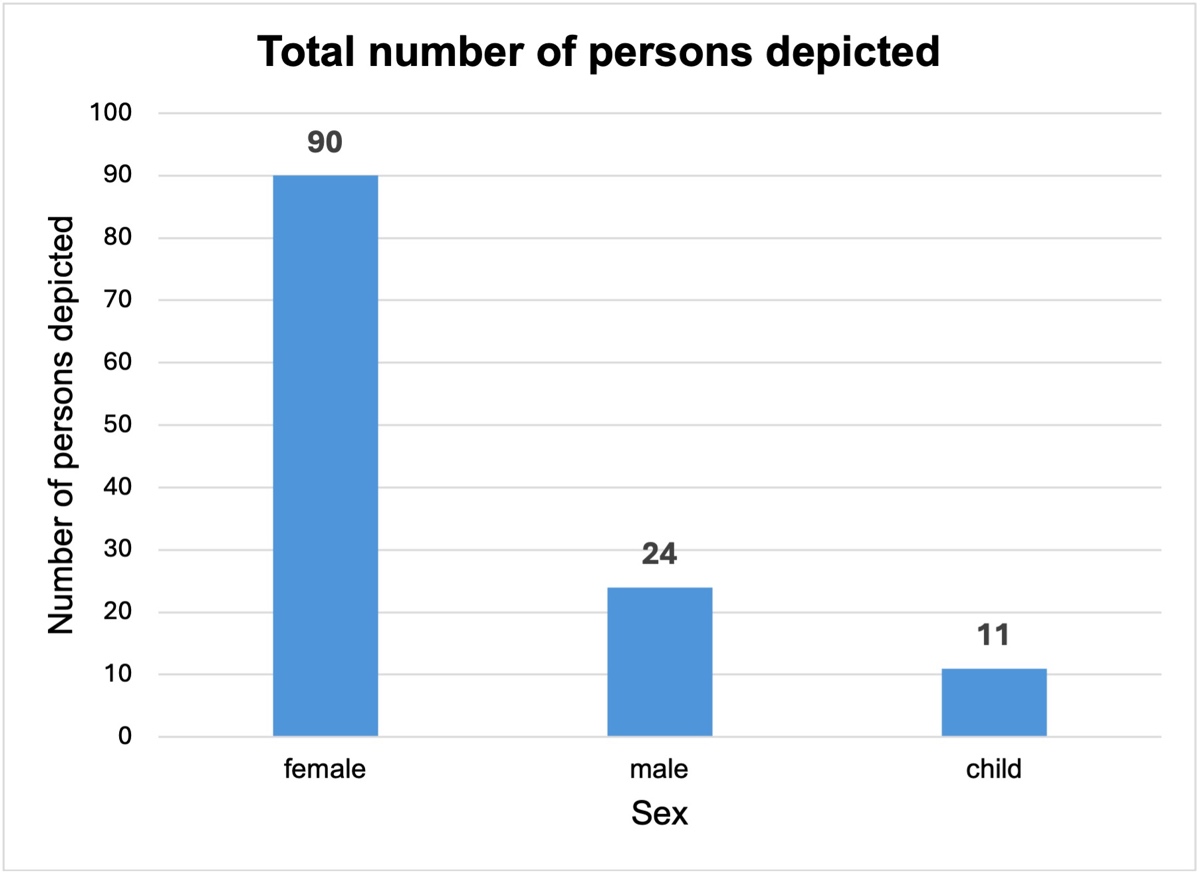


**Fig. S3** shows the total number of people shown in a bar chart


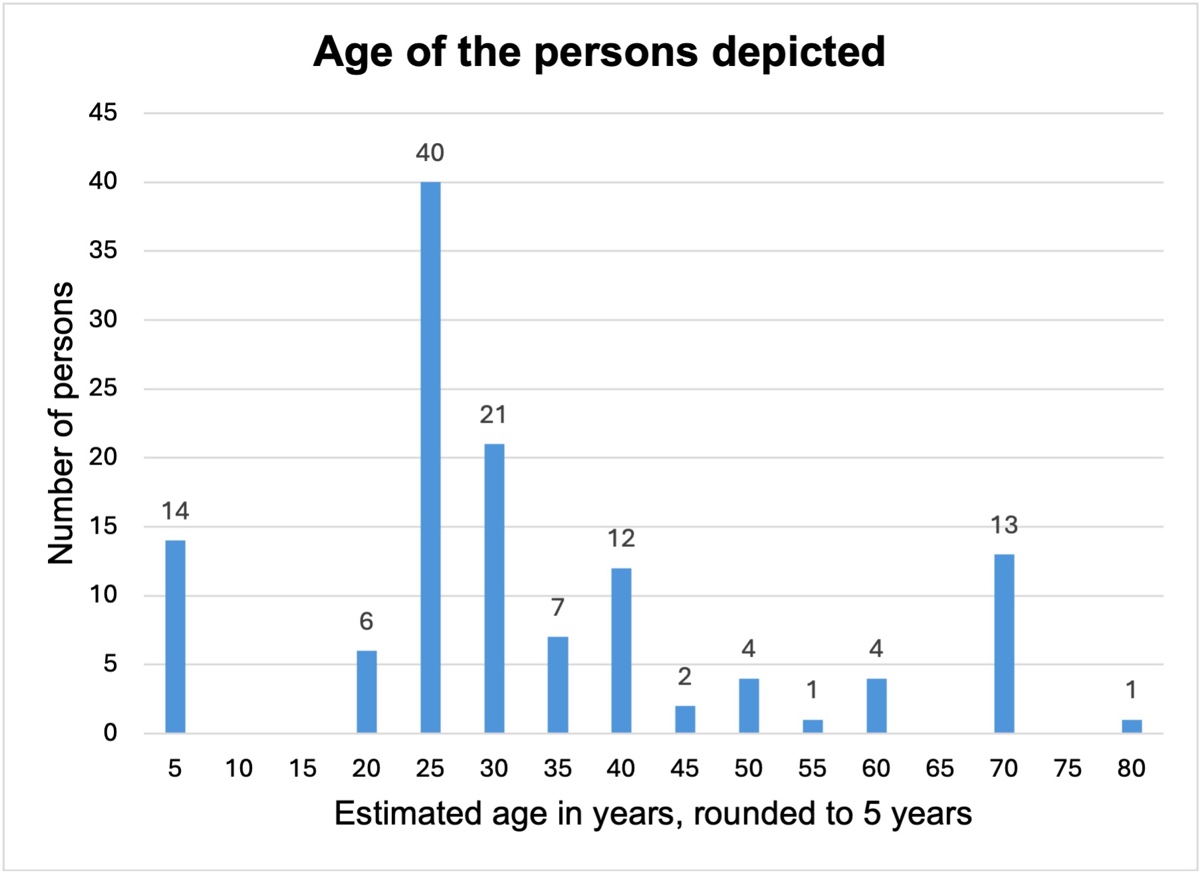


**Fig. S4** shows the age of the persons depicted in a bar chart


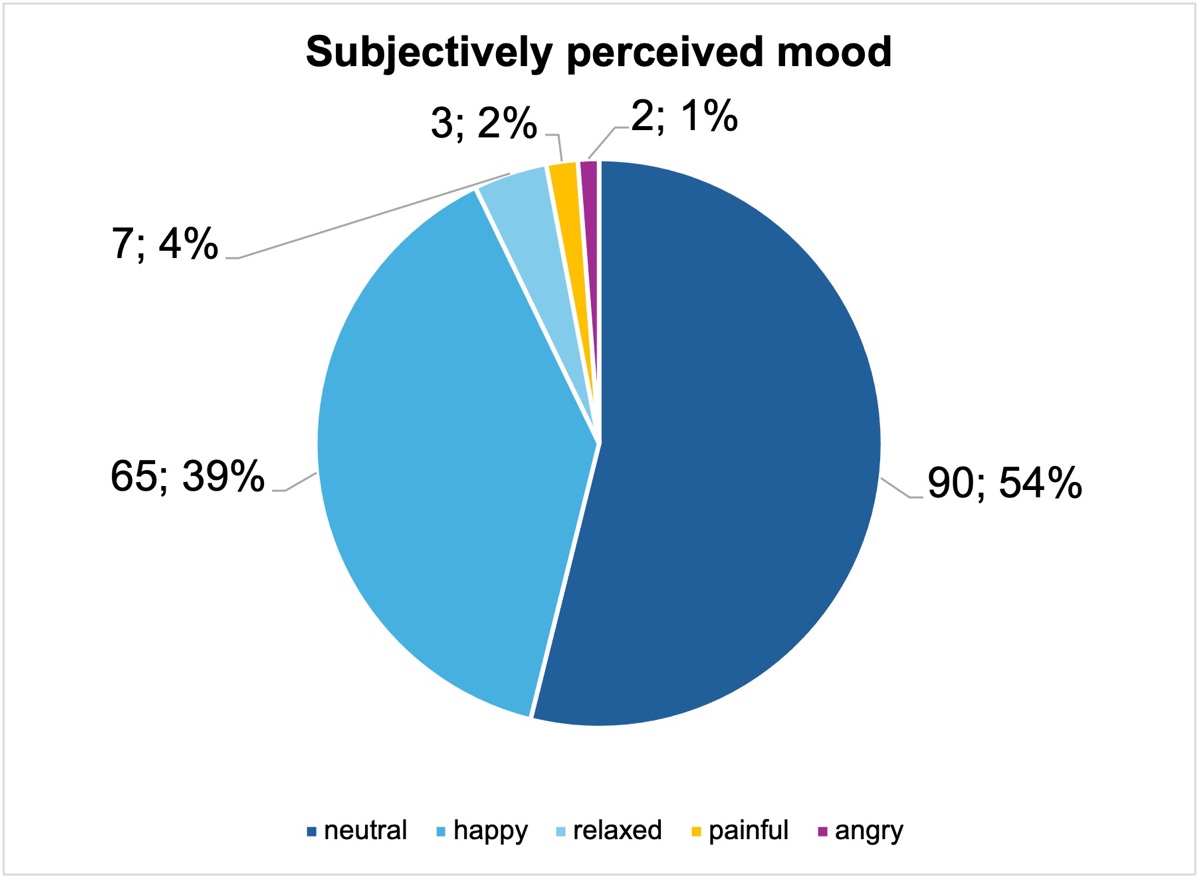


**Fig. S5** shows the subjectively perceived mood in the ads in a pie chart


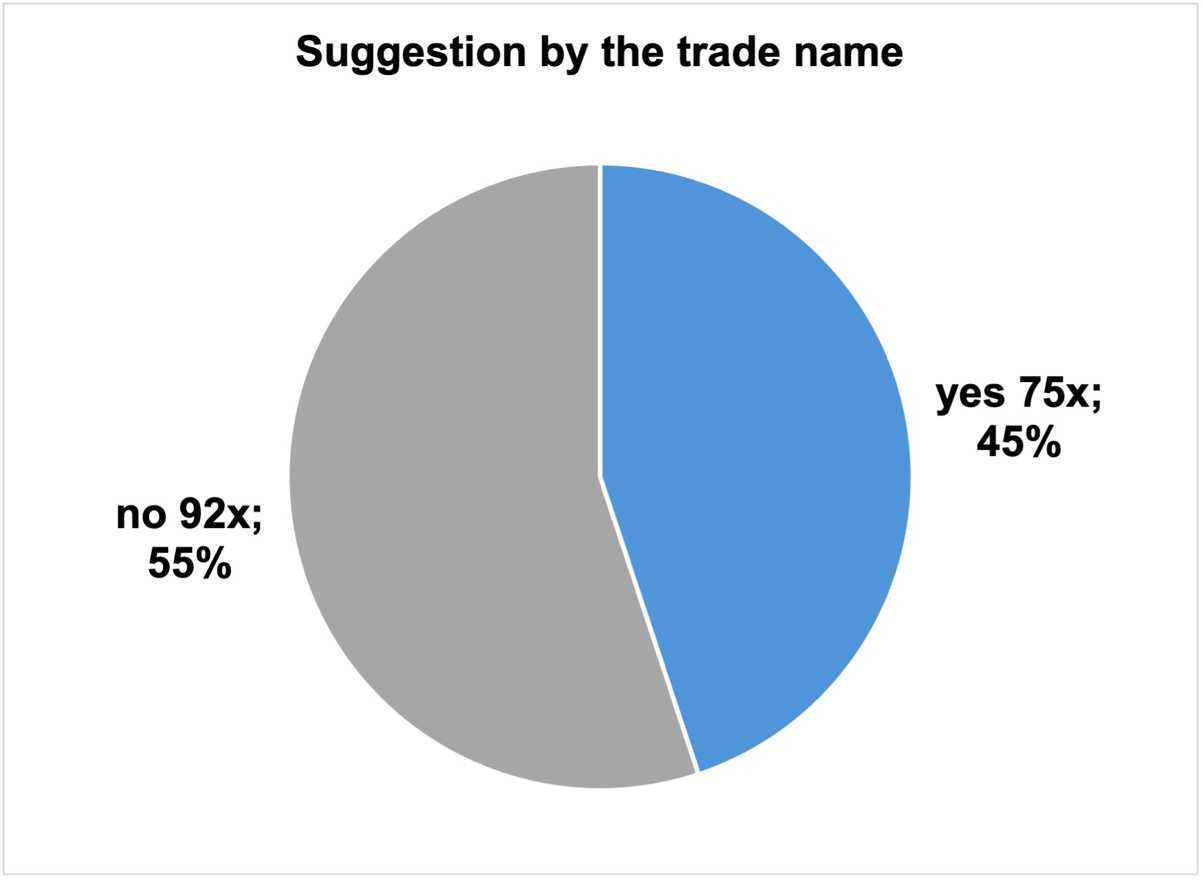


**Fig. S6** shows whether there is a suggestion by the trade name in a pie chart


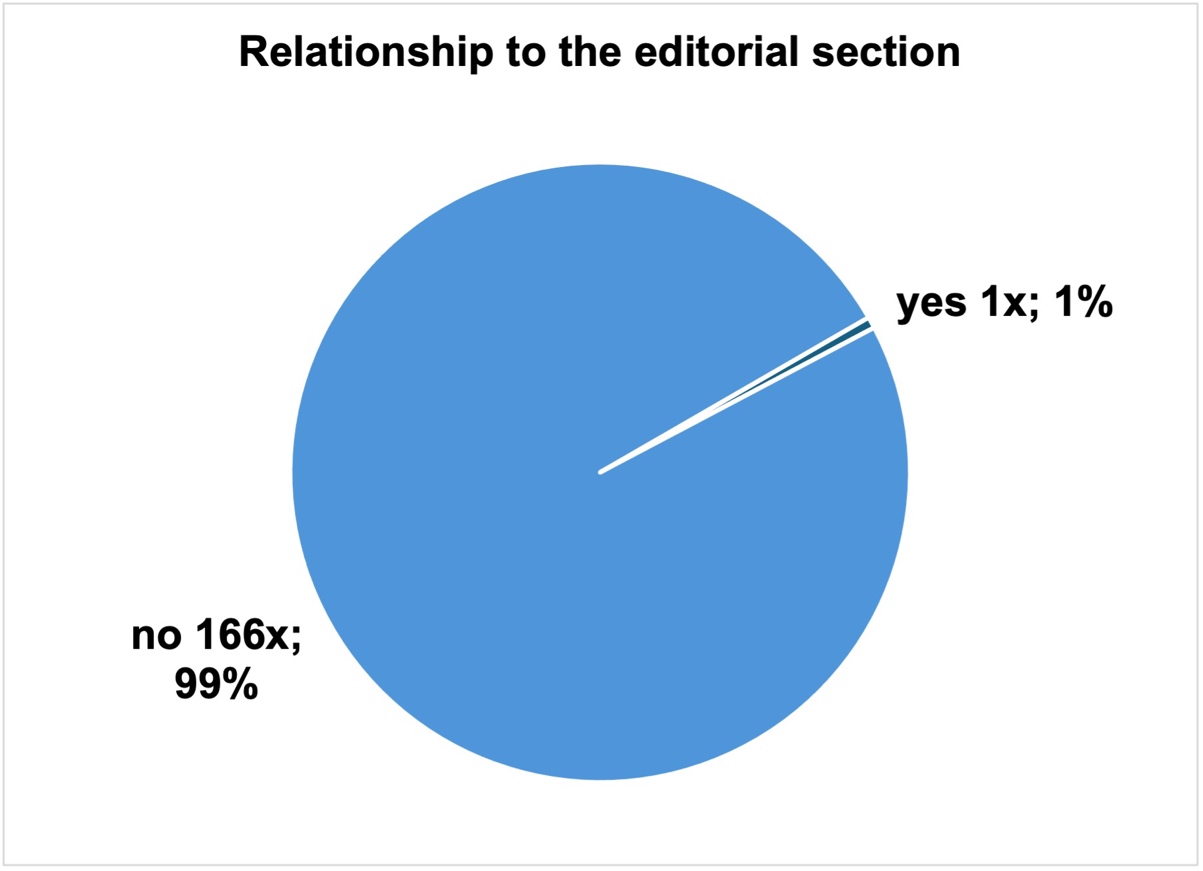


**Fig. S7** shows the relationship of the advertisements to the editorial section of the paper in a pie chart


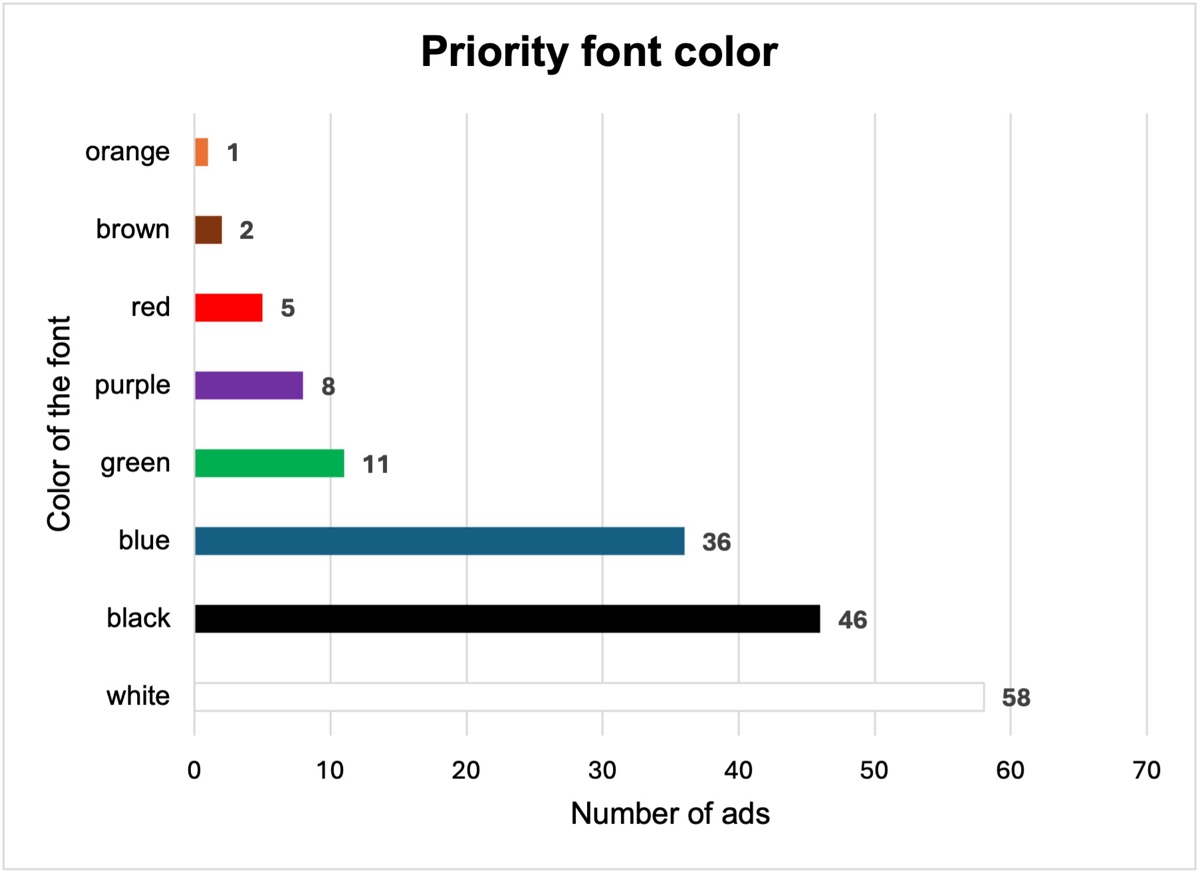


**Fig. S8 a** shows the priority color of the displays as absolute numbers in a bar chart


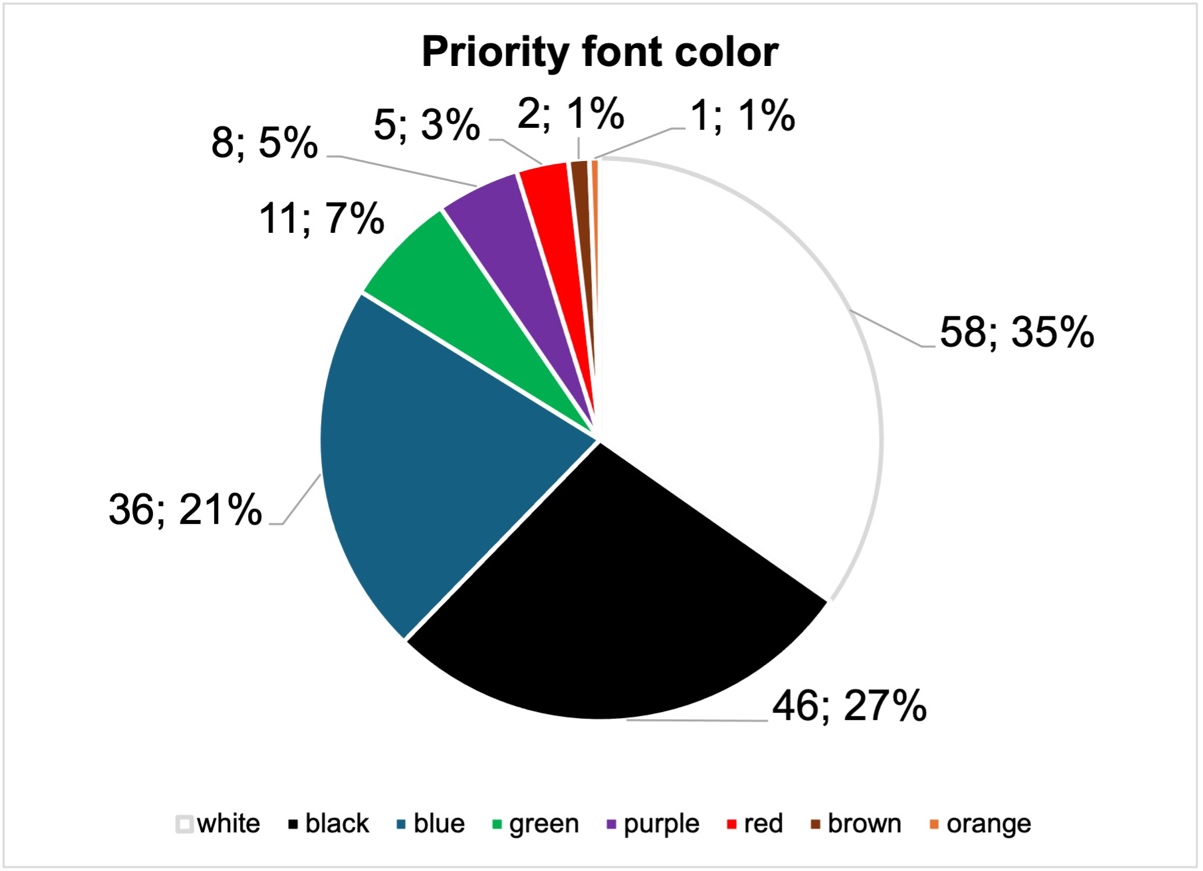


**Fig. S8 b** shows the priority color of the ads as absolute numbers and percentages in a pie chart


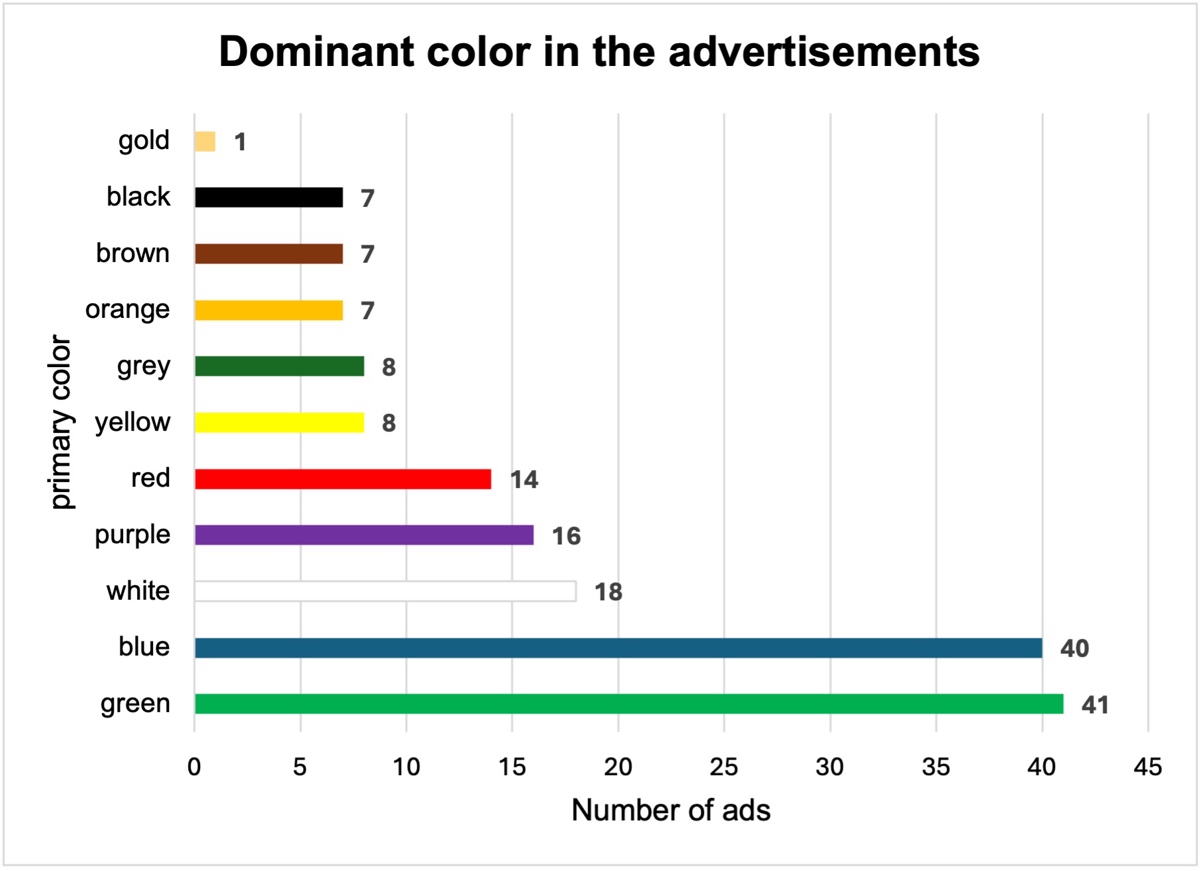


**Fig. S9 a** shows the dominant color in the displays in a bar chart


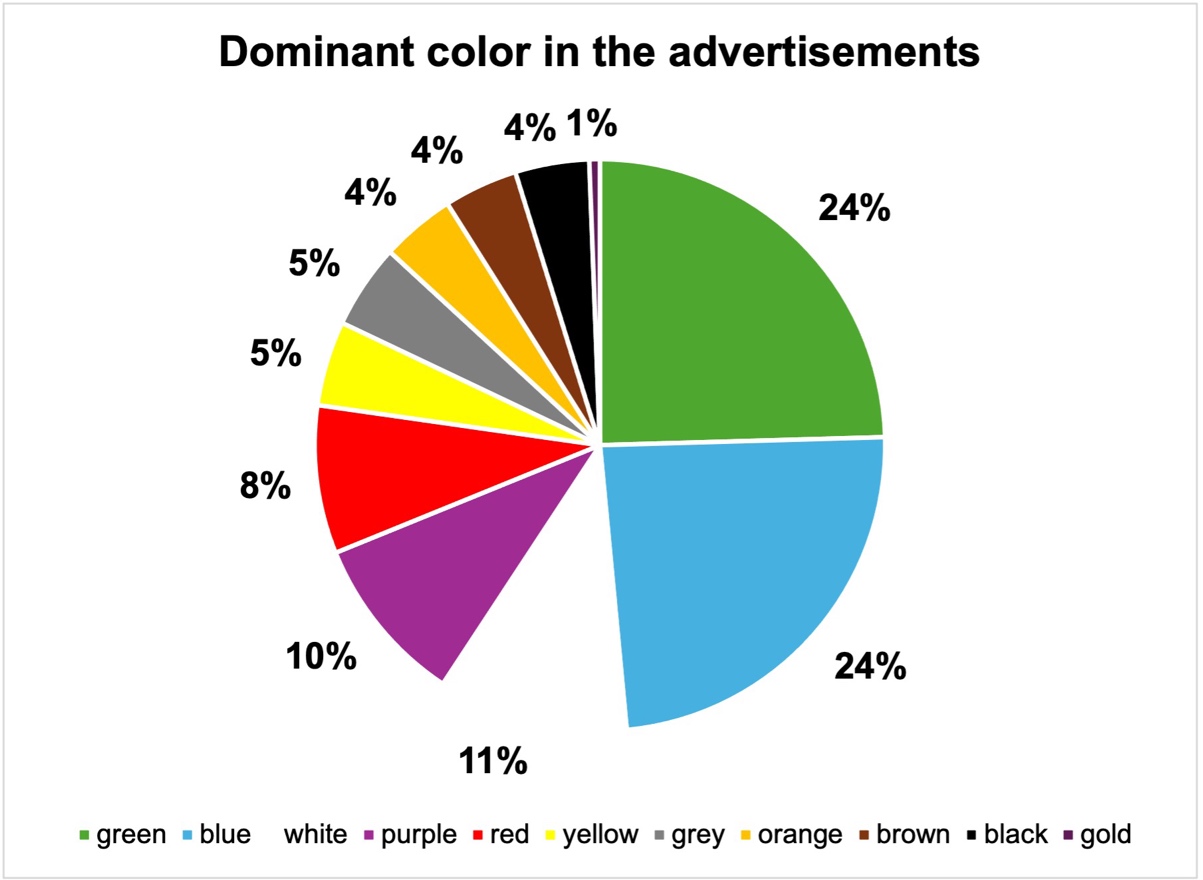


**Fig. S9 b** shows the dominant color in the displays as percentage in a pie chart


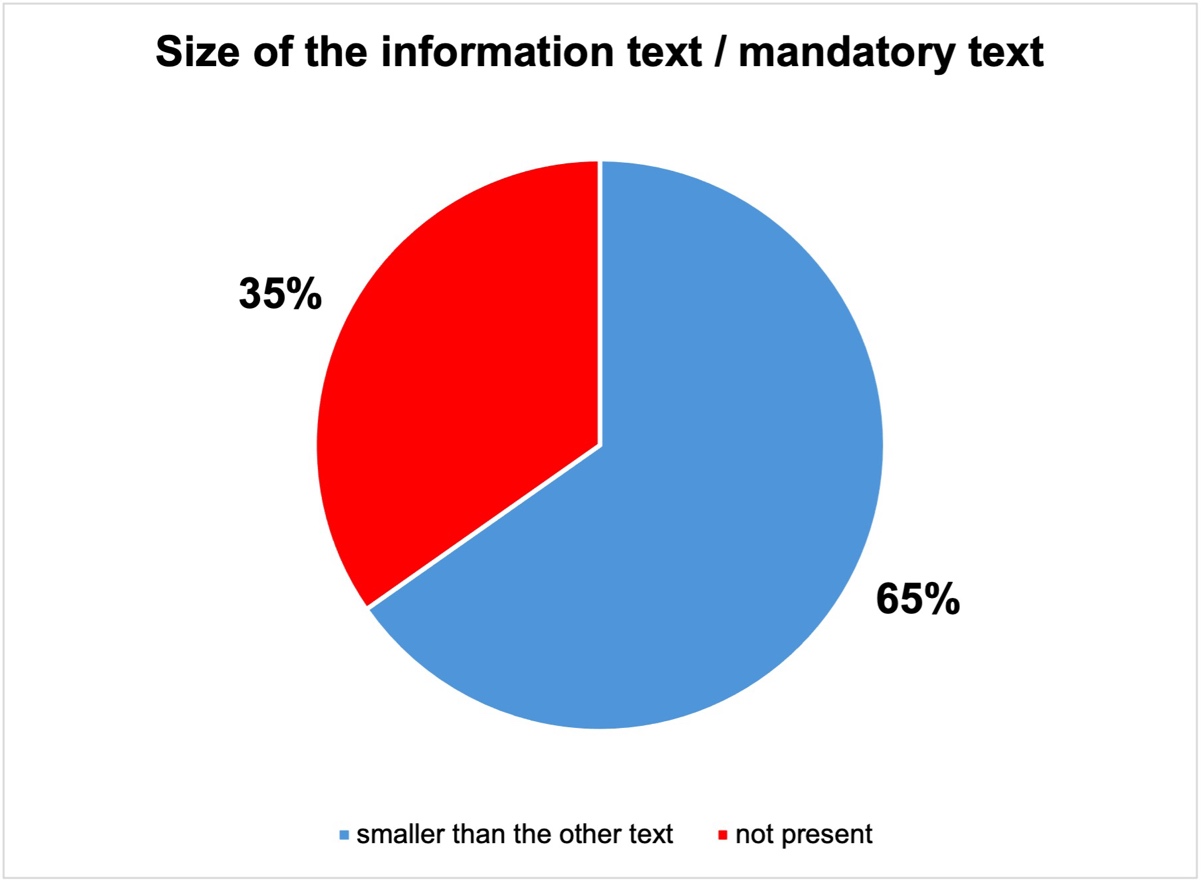


**Fig. S10** shows the size of the information text / mandatory text in the ads as percentage in a pie chart


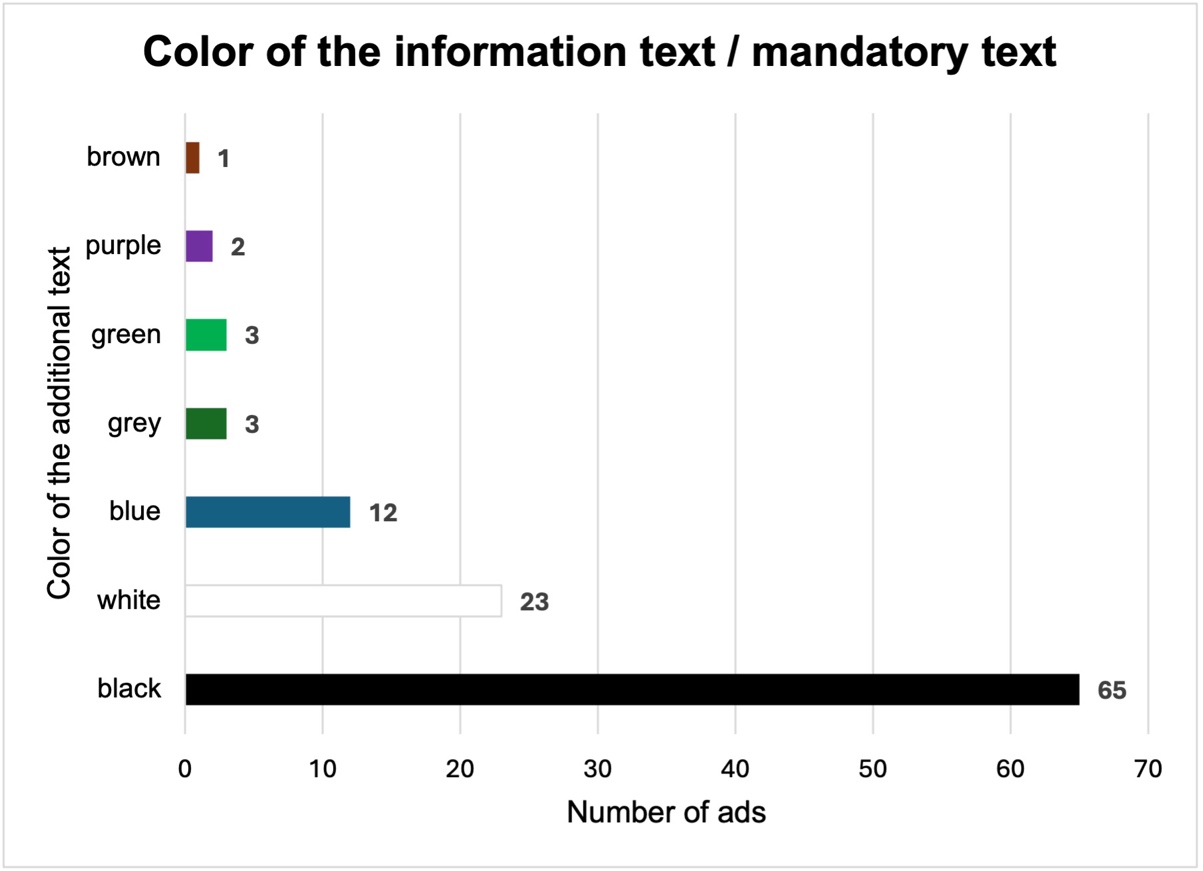


**Fig. S11 a** shows the color of the information text in absolute numbers in a bar chart


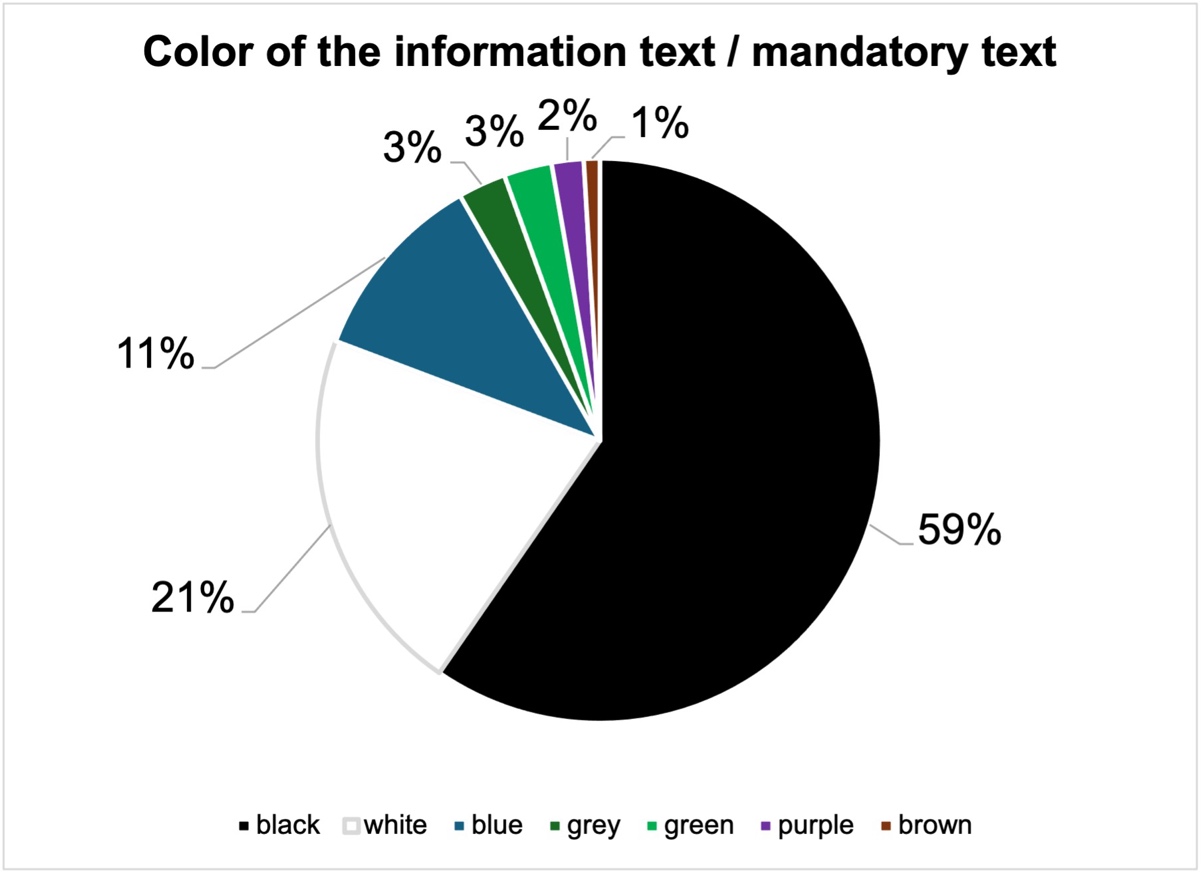


**Fig. S11 b** shows the color of the hint text as a percentage in a pie chart


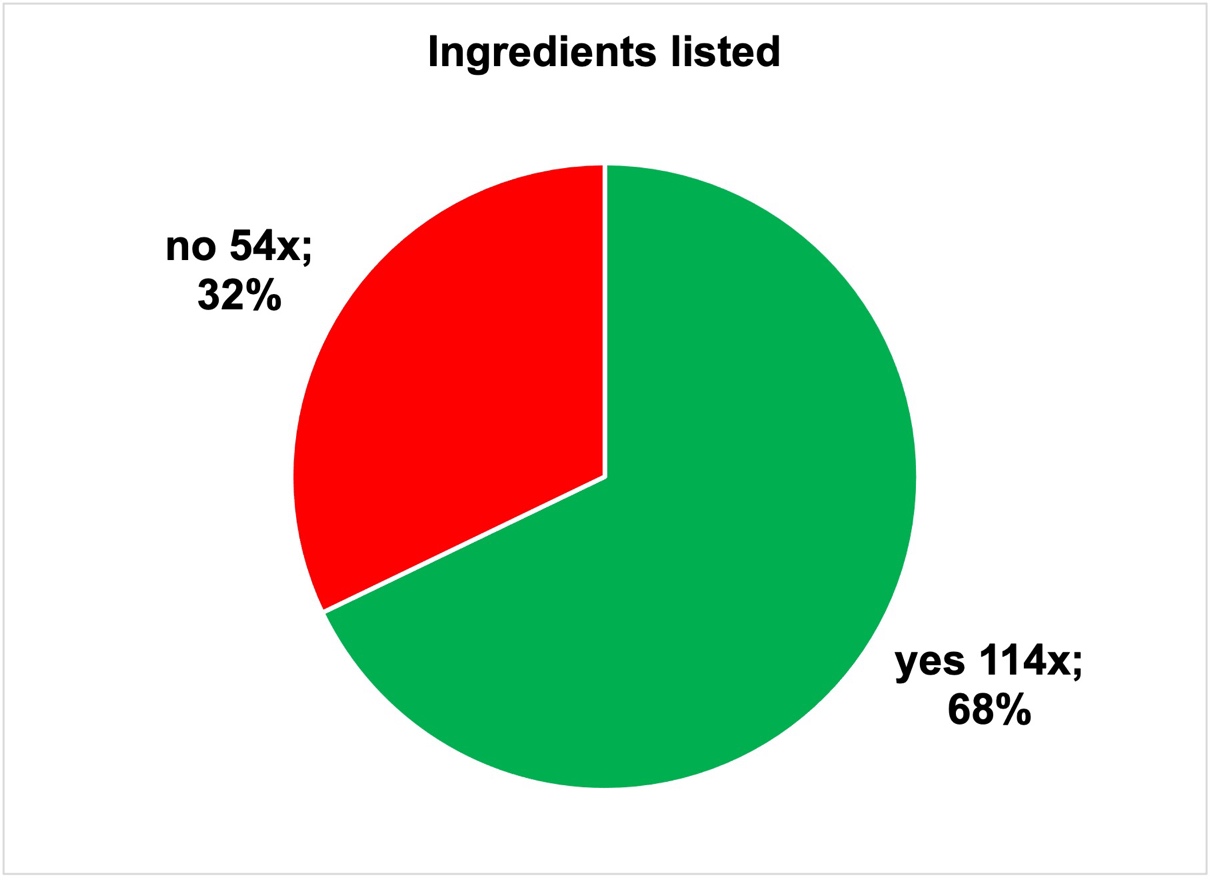


**Fig. S12** shows the ingredients in absolute numbers and percentages in a pie chart


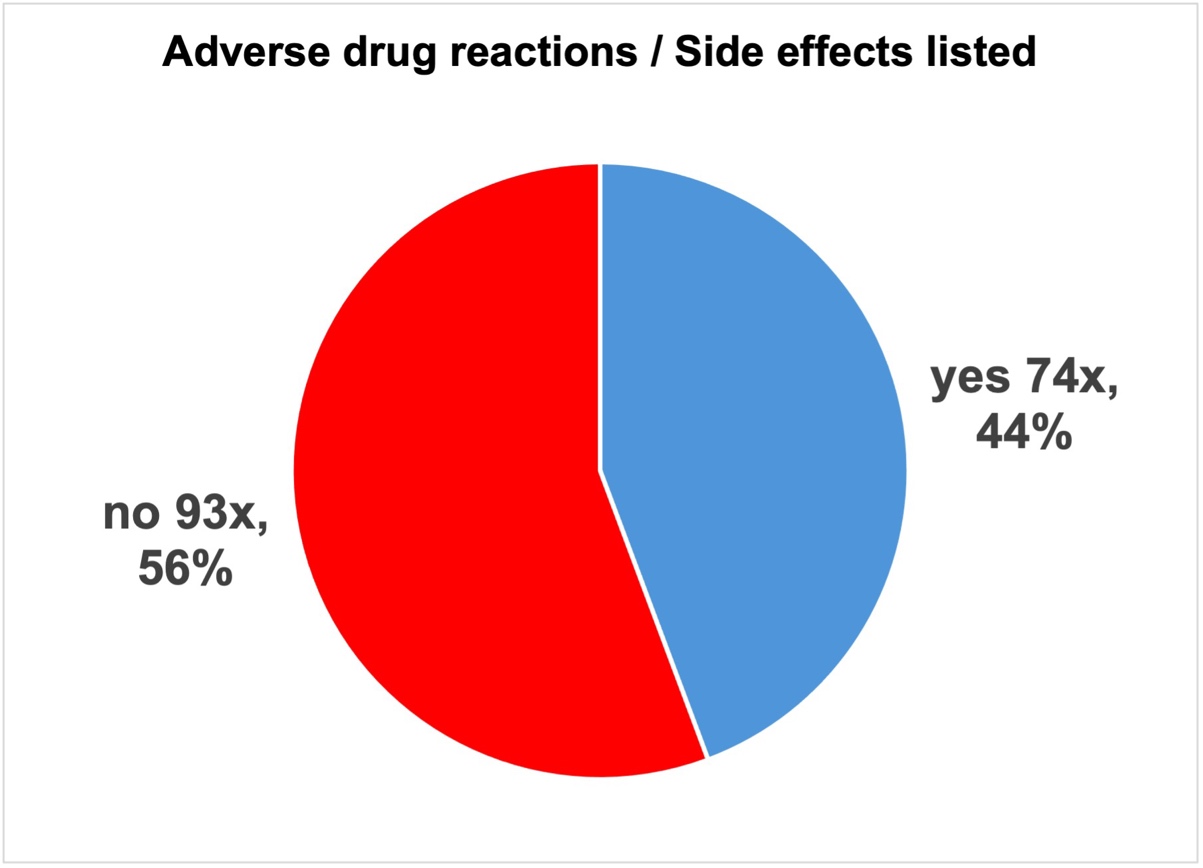


**Fig. S13** shows the indication of adverse drug reactions and side effects in a pie chart


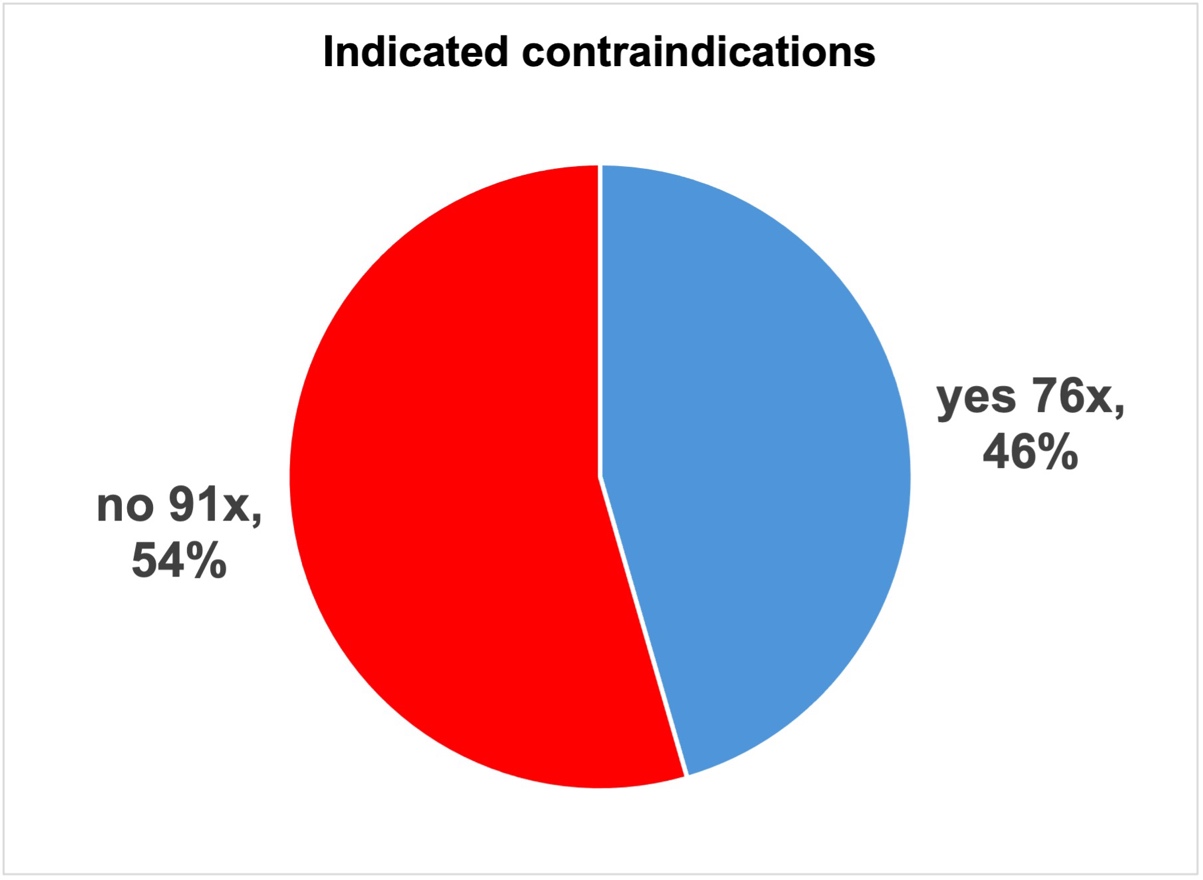


**Fig. S14** shows the listings of contraindications in a pie chart


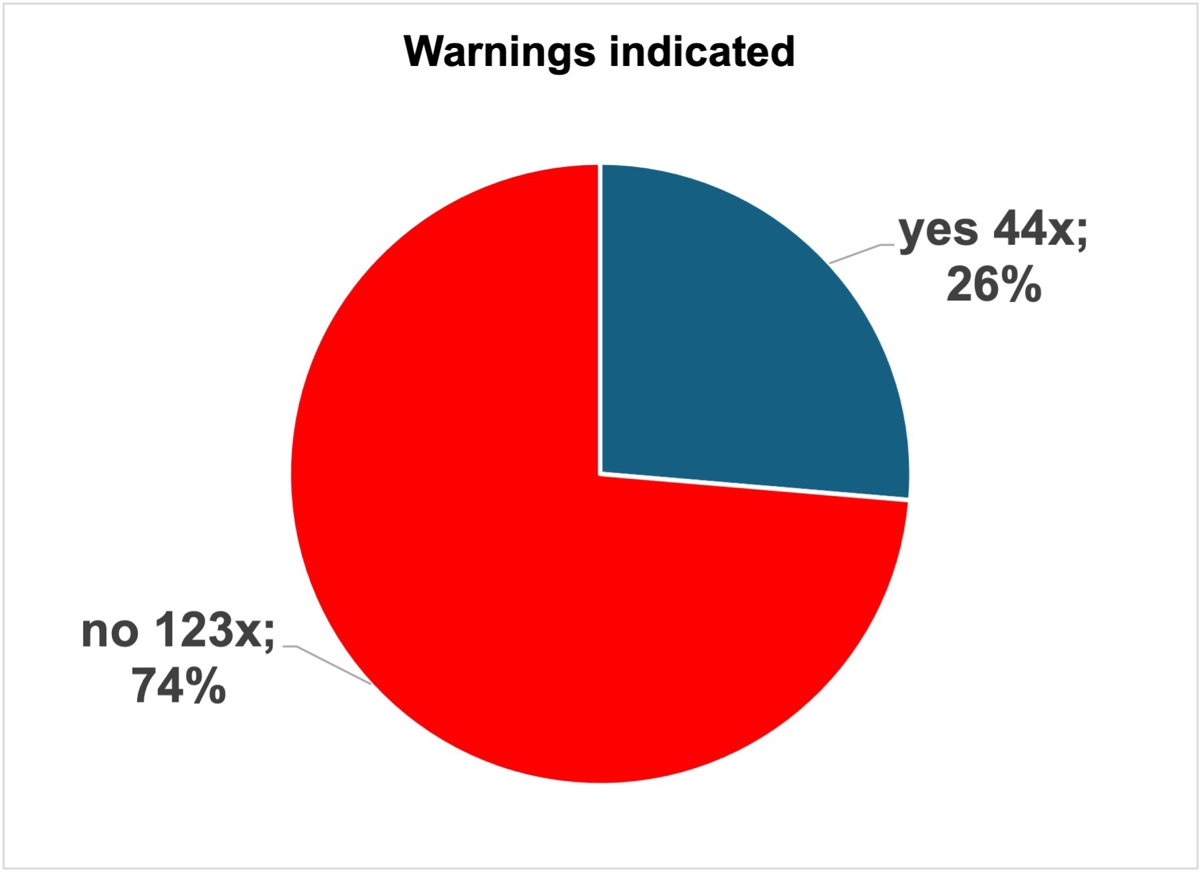


**Fig. S15** shows the indication of warnings in a pie chart


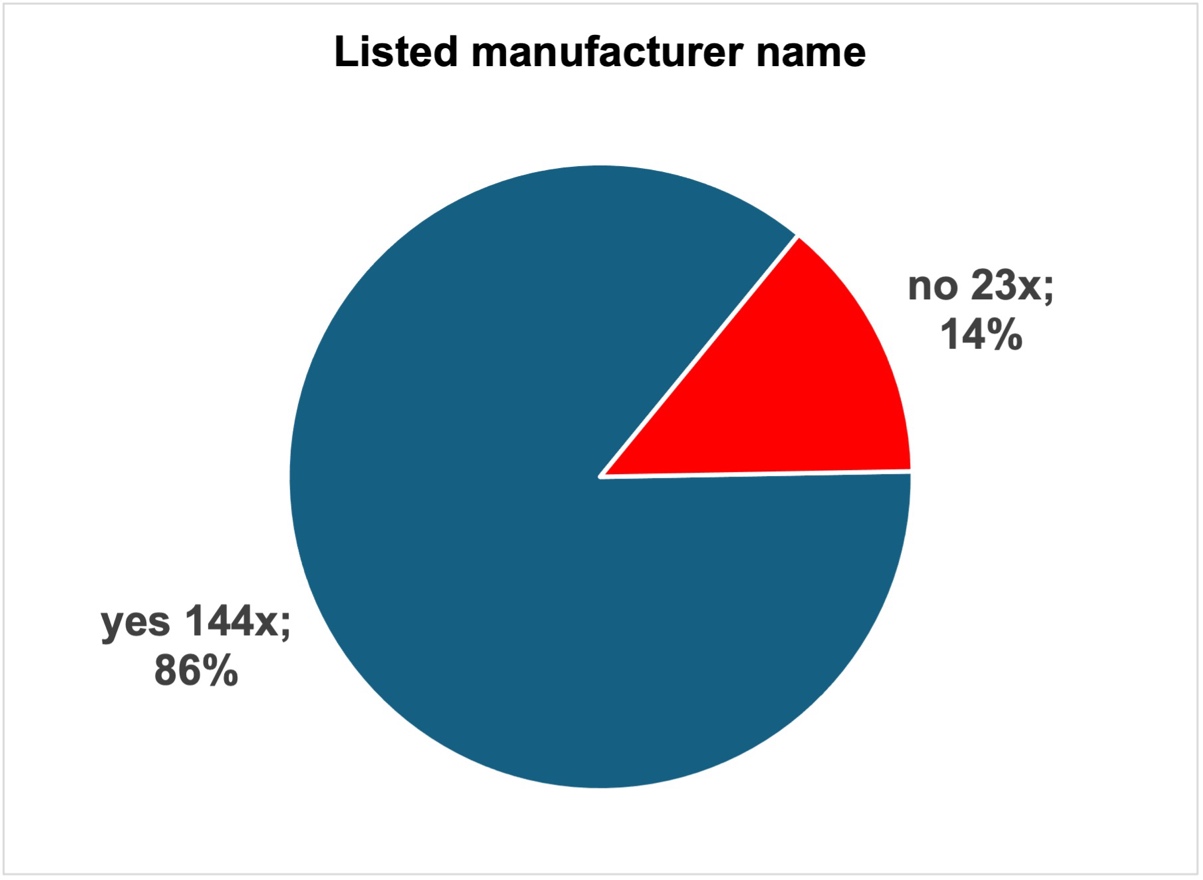


**Fig. S16** shows the listings of manufacturer name in absolute numbers and percentages in a pie chart


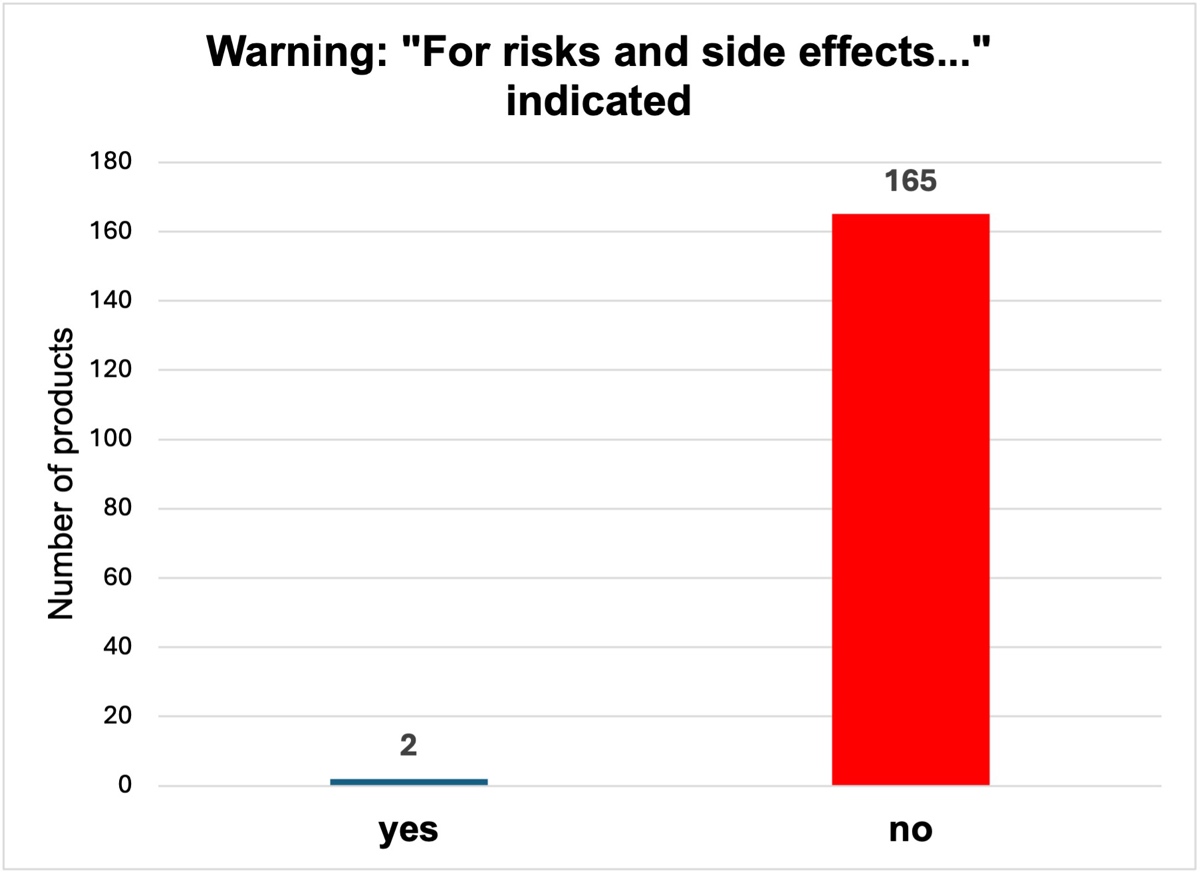


**Fig. S17** shows the warning "For risks and side effects..." as absolute numbers in a bar chart


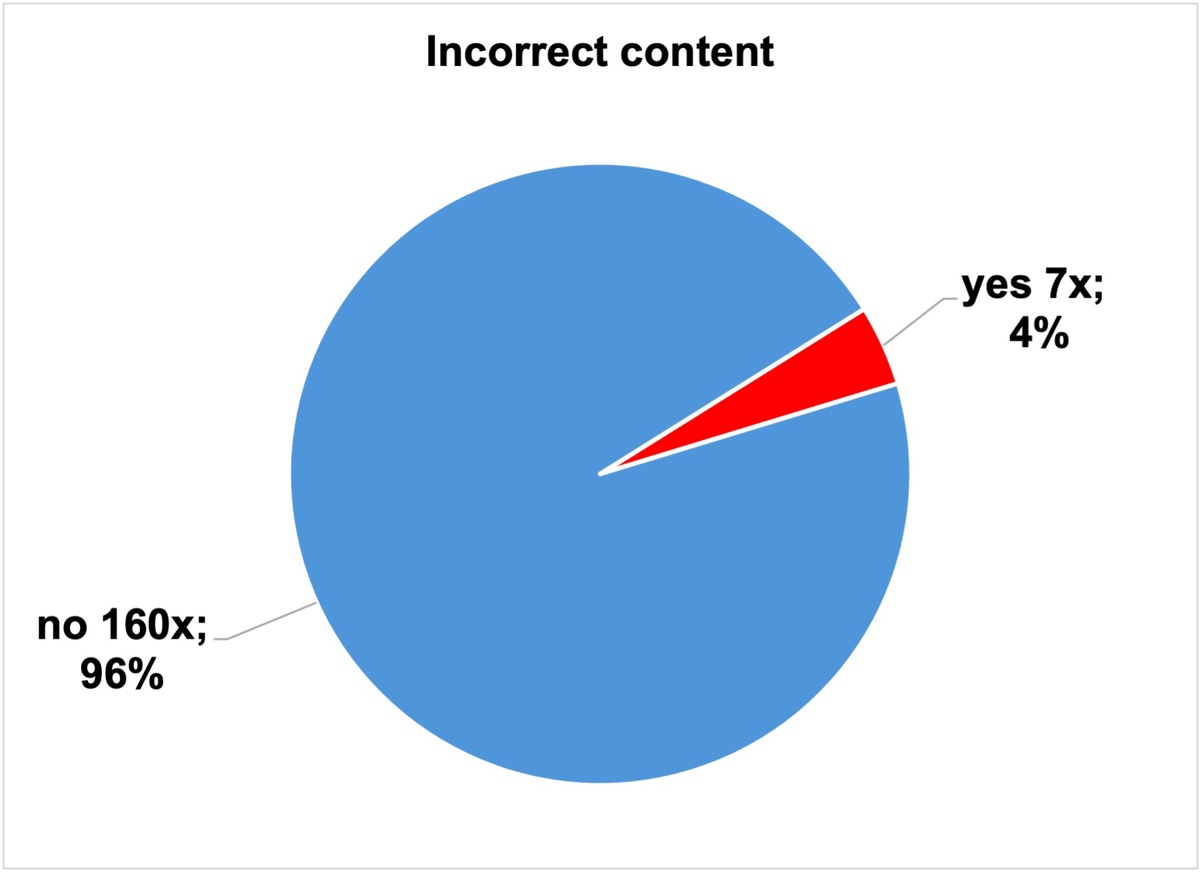


**Fig. S18** shows the indication of incorrect content in absolute numbers and percentages in a pie chart


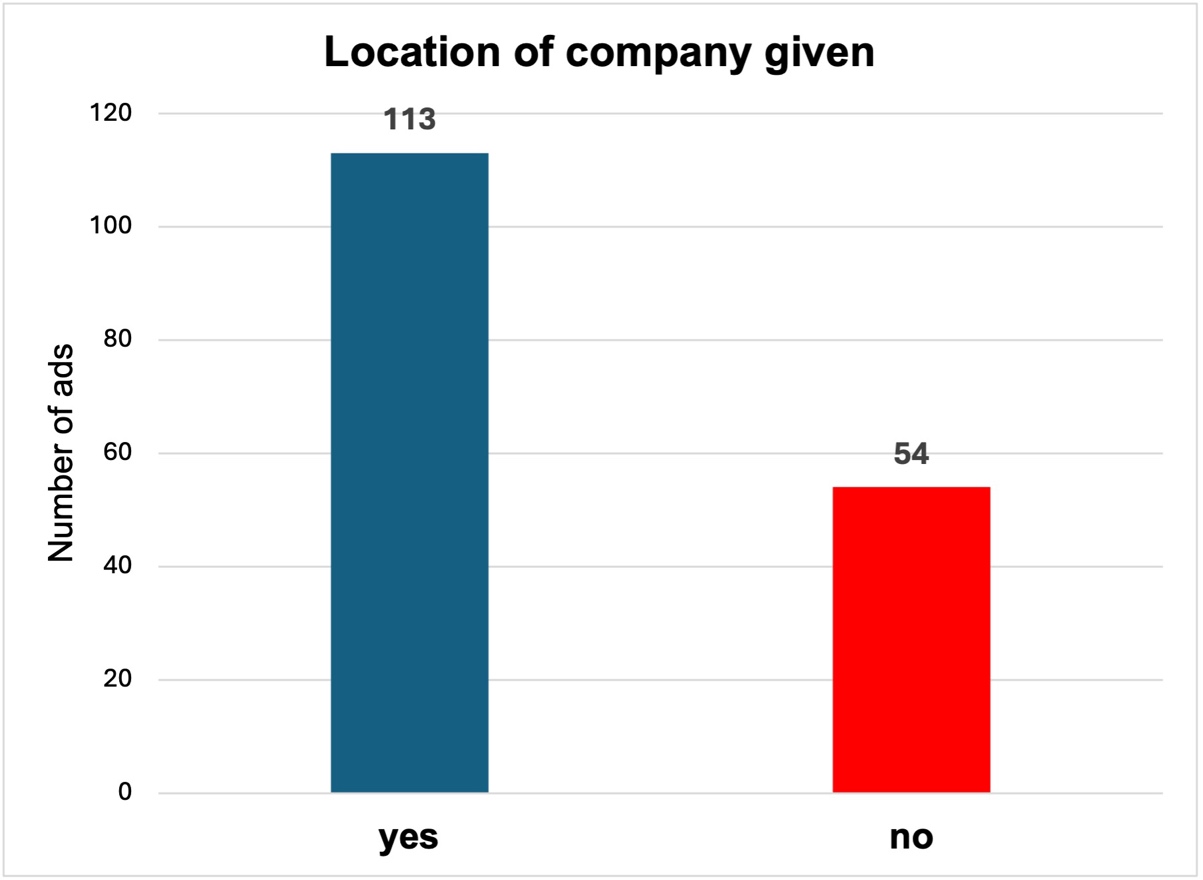


**Fig. S19** shows the location of the company as absolute figures in a bar chart
